# Olefin Ligand Metathesis for Colloidal Emissive Nanocrystals with Enhanced Stability and Photosensitivity

**DOI:** 10.1002/anie.202514802

**Published:** 2025-08-29

**Authors:** Seongbeom Yeon, Yoseph Kim, Abdessamad El Adel, Jaeyeong Ha, Seongkyu Maeng, Youngjo Kim, Ivan Infante, Himchan Cho

**Affiliations:** ^1^ Department of Materials Science and Engineering Korea Advanced Institute of Science and Technology (KAIST) 291 Daehak‐ro, Yuseong‐gu Daejeon 34141 Republic of Korea; ^2^ Department of Chemistry Chungbuk National University 1 Chungdae‐ro, Seowon‐gu Cheongju‐si Chungcheongbuk‐do 28644 Republic of Korea; ^3^ BCMaterials Basque Center for Materials, Applications, and Nanostructures UPV/EHU Science Park Leioa 48940 Spain; ^4^ Ikerbasque Basque Foundation for Science Bilbao 48009 Spain; ^5^ Graduate School of Semiconductor Technology School of Electrical Engineering Korea Advanced Institute of Science and Technology (KAIST) 291 Daehak‐ro, Yuseong‐gu Daejeon 34141 Republic of Korea

**Keywords:** Colloidal emissive nanocrystals, Direct optical patterning, Ligand chemistry, Olefin metathesis, Perovskite

## Abstract

High‐resolution patterning of colloidal perovskite nanocrystals (PNCs) is essential for next‐generation display technologies, yet conventional approaches relying on exogenous photosensitive ligands or additives often compromise optical properties and colloidal stability. Here, we present a nondestructive ligand modification strategy based on olefin metathesis, in which original oleic acid and oleylamine ligands are converted into metathesized ligands featuring dual anchoring groups and shortened chains. This structural transformation enhances colloidal stability through stronger chelation and reduced conformational entropy of possible ligand configurations. The removal of sterically hindering hydrocarbon chains exposes reactive alkene moieties, enhancing the photosensitivity of PNCs. The resulting metathesized PNCs (PNC‐M) exhibit excellent photoluminescence quantum yield (PLQY) retention (>93% after 3 weeks) and strong resistance to structural degradation under ambient conditions. Molecular dynamics simulations confirm the strengthened surface–ligand interactions in PNC‐M, consistent with the experimentally observed structural robustness. Furthermore, PNC‐M enables efficient direct optical lithography at substantially reduced UV doses via alkene polymerization and hydrothiolation, clearly outperforming pristine PNCs (PNC‐P). This strategy offers a general, nondestructive ligand engineering method for various emissive nanocrystals, including II–VI and III–V quantum dots, and facilitates high‐resolution lithography under reduced UV exposure by leveraging the enhanced photosensitivity imparted by olefin ligand metathesis.

## Graphical abstract

Olefin metathesis transforms original ligands on colloidal perovskite nanocrystals into metathesized ligands featuring dual anchoring groups, shortened chains, and exposed alkenes at the outermost ligand shell. The stronger binding and reduced conformational entropy enhance colloidal stability, while the exposed alkenes improve photosensitivity, enabling high‐resolution pattern formation under reduced UV exposure.

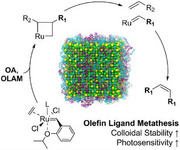

## Introduction

Colloidal lead halide perovskite nanocrystals (PNCs) have attracted considerable attention in optoelectronics and display technologies due to their exceptional optical properties, including high absorption coefficients, high photoluminescence quantum yield (PLQY), defect tolerance, and narrow emission spectra.^[^ ^1^, ^2^, ^3^, ^4^, ^5^ ^]^ The integration of PNCs into displays necessitates the formation of precisely defined pixel patterns through a patterning process. Among various patterning techniques, direct optical lithography has recently emerged as a simple and effective method for high‐resolution patterning of PNCs. This method exploits photo‐induced chemical reactions that modulate the solubility of PNCs, necessitating the introduction of photosensitivity into them.^[^ ^6^, ^7^, ^8^, ^9^ ^]^

Current strategies for photosensitization rely heavily on post‐synthetic treatments involving exogenous molecules, including i) ligand exchange with photosensitive molecules^[^ ^10^, ^11^, ^12^, ^13^, ^14^, ^15^ ^]^ and ii) the incorporation of additives such as crosslinkers,^[^ ^16^, ^17^, ^18^, ^19^, ^20^, ^21^ ^]^ photo‐acid generators,^[^ ^22^, ^23^, ^24^ ^]^ and photo‐base generators.^[^ ^25^ ^]^ However, these approaches often compromise the stability of PNCs due to the ionic nature of PNCs.^[^ ^26^ ^]^ The introduction of exogenous molecules can disrupt the delicate equilibrium between the native ligands and the PNC surface, with this equilibrium being highly composition‐dependent, thereby limiting the chemical window of ligand exchange. For instance, ligand exchange with amines can induce unintended phase transformations by extracting PNC components, such as converting CsPbBr_3_ to PbBr_2_‐depleted Cs_4_PbBr_6_.^[^ ^27^, ^28^, ^29^ ^]^ Meanwhile, the addition of highly acidic sulfonic acid can lead overly strong binding to PNCs, promoting surface etching and eventual degradation.^[^ ^30^ ^]^ Similarly, the incorporation of neutral amine, sulfoxide, thiophene, or thiol ligands exhibits opposite trends in the PLQY changes for CsPbBr_3_ and CsPbI_3_, indicating strong composition‐dependent effects.^[^ ^31^ ^]^ Such sensitivity to surface chemistry necessitates precise control over ligand‐surface interactions during patterning processes. In particular, ligand exchange‐based direct optical lithography requires the rational design of both binding groups and photosensitive moieties, as well as careful tuning of solubility and acidity, adding significant complexity to the process.

The use of additives presents an alternative approach, yet their photolysis often generates radicals that induce undesired side reactions,^[^ ^16^, ^17^, ^18^ ^]^ leading to degradation of PNC lattice or alteration of its ligand environment. Additionally, residual additive molecules can promote nanoparticle aggregation and induce nonuniform film morphologies, thereby deteriorating device performance. Consequently, strategies relying on exogenous molecules often compromise PNC stability, highlighting the need for an alternative method that simultaneously enhances photosensitivity and improves the stability of as‐synthesized PNCs.

**Figure 1:**
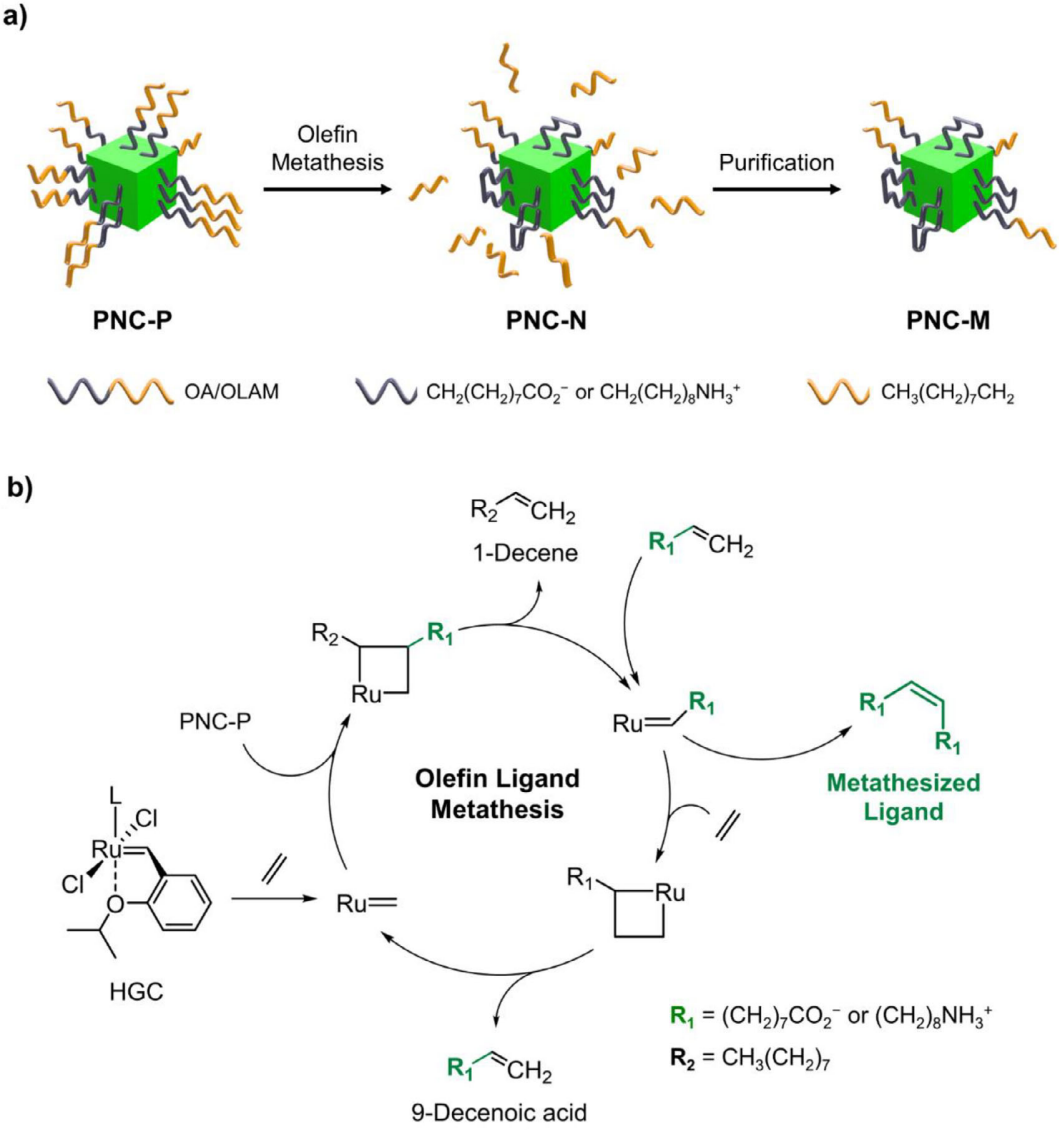
a) Schematic illustration of ligand modification via olefin metathesis in perovskite nanocrystals, showing the transformation from PNC‐P to PNC‐N and PNC‐M. b) Reaction mechanism of the olefin metathesis of the ligands in PNC‐P, where R_1_ represents either carboxylate or amine groups, and R_2_ is the alkyl chain.

Here, we present a nondestructive ligand modification strategy for PNCs via olefin metathesis, overcoming the challenges posed by exogenous photosensitive molecules. Our approach leverages ethylene‐mediated Hoveyda–Grubbs catalyst to selectively modify the internal olefin moieties of original ligands, namely oleic acid (OA) and oleylamine (OLAM), which are widely employed in PNC synthesis. Olefin ligand metathesis rearranges the alkene fragments in the original ligands, resulting in the formation of metathesized ligands. The produced metathesized ligands feature dual anchoring groups, reinforcing colloidal stability through stronger binding interactions with the PNC surface. Moreover, the removal of the outer hydrocarbon chains exposes sterically hindered olefin groups at the outermost region of the ligand shell, thereby enhancing their photosensitivity (Figure 1). As a result, PNCs modified with metathesized ligands (PNC‐M) demonstrated enhanced PLQY retention and superior structural stability under ambient conditions. In contrast, pristine PNCs (PNC‐P) exhibited significant structural instability over time, leading to significant PLQY degradation. These findings are supported by molecular dynamics (MD) simulations, which show that metathesized ligands ensure stronger binding, improved coverage, and suppressed structural fluctuations. Furthermore, the PNC‐M exhibited improved performance in direct optical lithography by facilitating alkene polymerization or hydrothiolation. This study demonstrates that the olefin metathesis enables both high‐resolution patterning and improved optical and colloidal stability of PNCs, addressing the issue induced by exogenous molecules. Moreover, this strategy can extend beyond PNCs to a broader range of colloidal emissive nanocrystals, including II–VI and III–V quantum dots (QDs), as long as they are capped with ligands containing internal olefinic protons.

## Results and Discussion

In pristine PNCs (PNC‐P) passivated by monodentate ammonium and carboxylate ligands, these surface ligands exhibit dynamic binding which facilitates proton exchange, reducing the colloidal stability of PNCs (Figure 1a).^[^ ^32^ ^]^ Additionally, the internal C9═C10 double bond can participate in photochemical reactions, but the outer C11–C18 hydrocarbon chain sterically hinders the inner alkene group, compromising photosensitivity. To improve the colloidal stability and enhance the photosensitivity of PNCs without causing potential degradation, ligand modification via olefin metathesis was designed and performed. Olefin metathesis is a chemical reaction that redistributes alkene fragments by breaking and reforming C═C double bonds. This reaction is catalyzed by organometallic complexes, such as the Schrock catalyst, which uses tungsten or molybdenum as its central metals, and the Grubbs catalyst, which uses ruthenium (Ru).^[^ ^33^ ^]^ Since the central metals in the Schrock catalyst exhibit high electrophilicity toward the lone pairs of electrons on oxygen and nitrogen in ligands,^[^ ^33^ ^]^ the Grubbs catalyst was introduced for ligand modification. We specifically selected second‐generation Hoveyda–Grubbs catalyst (HGC), which has phenol derivative replacing phosphine, due to its high air and moisture stability.^[^ ^34^ ^]^

To effectively remove the outer hydrocarbon chains that sterically hinder the photochemical reactions between alkene moieties on adjacent PNCs, we employed ethylene, the smallest nonsubstituted alkene molecule. The injected ethylene forms a complex with HGC (denoted as Ru═), which redistributes alkene moieties in the PNC ligands, leading to the formation of a disubstituted metallacyclobutane intermediate through direct [2+2] cycloaddition (Figure 1b). The cross‐metathesis reaction between ethylene and the olefin ligand generates ethenolyzed ligands with a single anchoring group (H_2_C═CH–R_1_; R_1_ = (CH_2_)_7_CO_2_^−^ or (CH_2_)_8_NH_3_^+^; 9‐decenoic acid or 9‐decen‐1‐amine), along with 1‐decene as a noncoordinating product. Meanwhile, the self‐metathesis reaction between ethenolyzed ligands results in the formation of metathesized ligands (R_1_–HC═CH–R_1_; R_1_ = (CH_2_)_7_CO_2_^−^ or (CH_2_)_8_NH_3_^+^) featuring dual anchoring groups, which potentially adopt a bidentate coordination with PNC surface. Since olefin metathesis is an equilibrium‐driven process,^[^ ^35^ ^]^ the ethylene concentration was deliberately increased to shift the equilibrium toward the desired ligand modification. To drive the forward reaction in accordance with *Le* Chatelier's principle, we injected ethylene gas at high pressure (10 bar) into the reaction vessel (Figure S1). Following olefin ligand metathesis, not only modified ligands, but also excess ethylene, catalyst, and noncoordinating byproducts remained in the PNCs (PNC‐N). Therefore, a purification process was conducted to remove these impurities, yielding PNCs capped with metathesized ligands (PNC‐M) (Figure 1a).

### Ligand Modification via Olefin Metathesis

**Figure 2:**
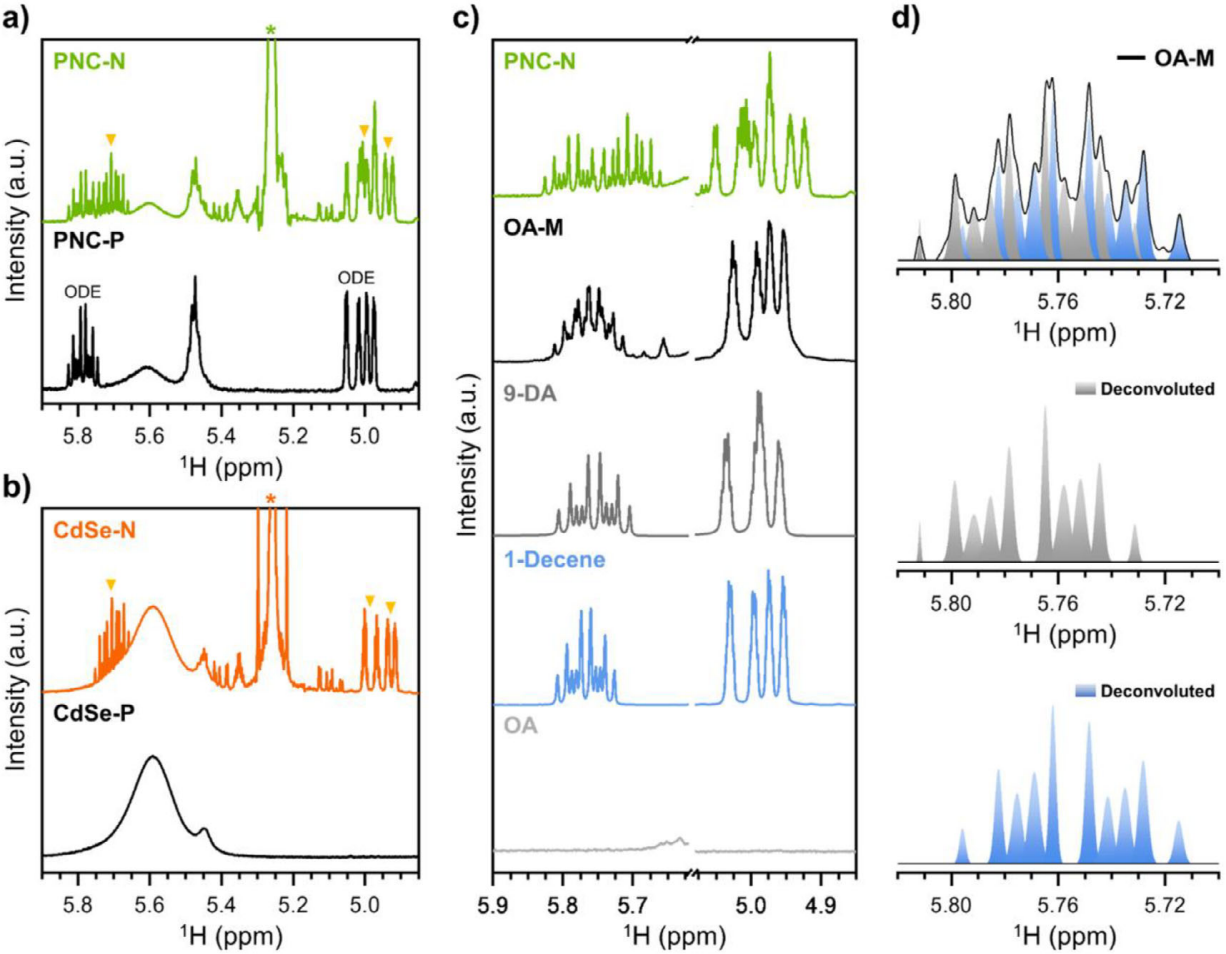
a,b) ^1^H NMR spectra of a) PNC‐P and PNC‐N, b) CdSe‐P and CdSe‐N, describing alkene protons. c) ^1^H NMR spectra of PNC‐N, metathesized oleic acid (OA‐M), 9‐decenoic acid (9‐DA), 1‐decene, and OA, describing vinylic protons. d) Deconvoluted ^1^H NMR spectrum of OA‐M corresponding to vicinal vinylic protons of the terminal alkene. The orange triangles and asterisk symbols indicate terminal alkene protons and toluene‐*d*_8_, respectively.

The effects of olefin metathesis on the ligand environments are systematically investigated using nuclear magnetic resonance (NMR) spectroscopy. The ^1^H NMR spectrum of PNC‐P exhibits peaks near 5.00 and 5.80 ppm, attributed to the terminal *sp^2^*‐H of 1‐octadecene (ODE), used as a synthetic solvent (Figures 2a and S2). Additionally, the peak near 5.60 ppm corresponds to internal *sp^2^*‐H from ligands bound to the PNC surface, whereas the peak at 5.46 ppm is assigned to free ligands.^[^ ^32^ ^]^ Individual treatment of PNCs with either HGC or ethylene resulted in no discernible spectral changes (Figure S3), suggesting that neither reactants independently alter the ligand environment of PNCs. In contrast, the simultaneous addition of both reactants induced notable spectral changes. The ^1^H NMR spectrum of PNC‐N revealed new peaks in the 4.8–5.8 ppm region after the reaction, indicating the formation of new alkene species (Figure 2a). These newly formed alkene species originate from the PNC ligands, as cross‐metathesis between ODE and ethylene cannot produce such terminal alkene species (See Supporting Information Text S1, Figures S4–S6).

However, the possibility of ODE participation in metathesis reaction through its C17═C18 moiety, and the coexistence of OA and OLAM ligands complicate the assignment of spectral changes. To address this, we introduced a model system using cadmium selenide (CdSe) quantum dots (QDs) exclusively passivated with OA ligands (CdSe‐P). CdSe QDs are suitable for this purpose, as the strong coordination between OA and the CdSe surface enables multiple purification steps to remove residual ODE without significant degradation. This allows us to clearly attribute the observed spectral changes to ligand modifications, providing direct evidence of olefin ligand metathesis (Figure 2b). In CdSe‐P, only OA ligands underwent metathesis, producing CdSe QDs with modified ligands and noncoordinating by‐products (CdSe‐N). The newly generated species in CdSe‐N displayed ^1^H NMR spectral features identical to those of PNC‐N (see more details in the Terminology Note, Figure S7; Tables S1, S2), indicating that the same reactions originate from olefin ligands in both systems. In particular, both PNC‐N and CdSe‐N exhibited 13 symmetric peaks in the vicinal vinylic proton region of terminal alkene, attributed to overlapping signals from two distinct alkene species. Peak deconvolution analysis confirmed the presence of these distinct alkene species (Figure S8).

Identifying the alkene products provides direct evidence of olefin ligand metathesis. To validate this finding, a cross‐metathesis reaction between OA and ethylene was performed (Figure S9). The ^1^H NMR spectrum of metathesized oleic acid (OA‐M) confirms the formation of new alkene species (Figures 2c and S10). The peak around 5.75 ppm in OA‐M strongly suggests two structurally distinct terminal alkene species (Figure 2d). Since only 1‐decene and 9‐decenoic acid (9‐DA) can form as terminal alkene species through cross‐metathesis of OA with ethylene, the ^1^H NMR spectrum of PNC‐N was compared with the spectra of 1‐decene and 9‐DA, and spectral match was observed (Figure S11). Through this control experiment, two alkene species in PNC‐N and CdSe‐N were assigned to 1‐decene and 9‐DA, respectively, providing direct evidence of olefin ligand metathesis.

**Figure 3:**
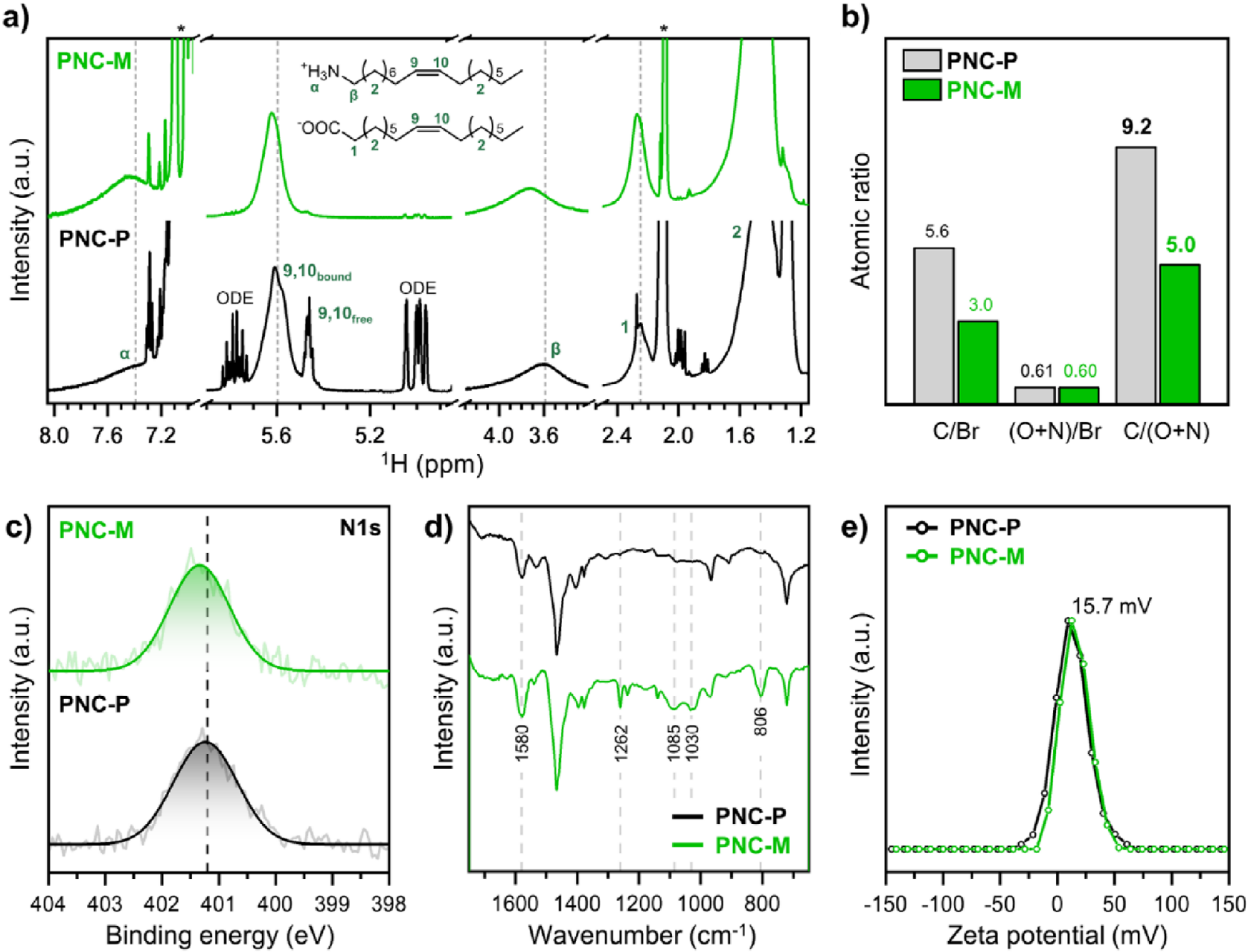
Characterization of modified ligands through olefin metathesis. a) ^1^H‐NMR spectra showing the changes in chemical shifts after metathesis reaction in PNCs. b) Relative XPS atomic ratios of PNCs to ligands. c) XPS spectra of N1s orbital in PNC‐P and PNC‐M. d) FTIR spectra and e) zeta potential values of PNC‐P and PNC‐M.

Subsequently, the ligand environment of metathesized PNCs (PNC‐M) was analyzed and compared with that of PNC‐P after the removal of non‐coordinated species (Figure 3a). In the ^1^H NMR spectrum of PNC‐M, peak broadening and downfield shifts were observed for –CH_2_–NH_3_^+^ (3.6 and 7.4 ppm), internal *sp* ^2^‐H (5.60 ppm) and –CH_2_–COO^−^ (2.2 ppm). These spectral changes suggest enhanced ligand binding at the PNC surface, likely resulting from bidentate configuration that strengthens chelation, as well as reduced conformational entropy due to shortened chain length.^[^ ^36^ ^]^ Stronger surface‐ligand interactions restrict molecular rotational dynamics, making dipolar interactions less efficiently averaged. This corresponds to a decrease in the transverse relaxation time (T_2_), resulting in pronounced NMR line broadening.^[^ ^37^ ^]^ In addition, the observed downfield shifts are closely related to changes in chemical shielding effects. Stronger ligand binding perturbs the local electronic environment near the PNC surface, leading to reduced electron shielding and downfield chemical shift.^[^ ^37^, ^38^, ^39^ ^]^ The –CH_2_–NH_3_^+^ moiety, being highly sensitive to local electrostatic environments on PNC surface, clearly exhibits these changes. Therefore, spectral broadening and downfield shifts in PNC‐M reflect stronger ligand coordination resulting from olefin metathesis. Nuclear overhauser effect spectroscopy (NOESY) further supports tight ligand binding in PNC‐M. In the NOESY spectrum, olefin protons exhibit significant spatial correlations between adjacent ligands (Figure S12), confirming their binding to the PNC surface. This spatial correlation is characteristic of surface‐bound ligands rotating together with the PNCs, resulting in restricted motion and increased rotational correlation time. Under these conditions, negative NOE cross‐peaks are observed,^[^ ^40^, ^41^, ^42^ ^]^ as shown in Figure S12. These NMR analyses provide evidence for the strong coordination of metathesized ligands at the PNC surface.

The ligand environment of PNC‐M was further analyzed using X‐ray photoelectron spectroscopy (XPS), which quantified the surface atomic ratio of Br to ligands in PNCs.^[^ ^43^ ^]^ XPS revealed a significant decrease in the Br‐to‐ligand tail (hydrocarbon chain) ratio, while the Br‐to‐ligand head (binding group; COO^−^ of OA, and NH_3_^+^ of OLAM) ratio remained unchanged (Figure 3b). The reduced Br‐to‐C ratio strongly suggests removal of the outer C11–C18 hydrocarbon chains, rather than loss of Br atoms. In contrast, the unchanged Br‐to‐head ratio confirms the preservation of surface‐binding groups. This finding shows that our olefin ligand metathesis strategy selectively restructures the ligand backbone while retaining their surface anchoring to PNCs. XPS also supported enhanced ligand binding of metathesized ligands, evidenced by the higher N1s binding energy in PNC‐M relative to PNC‐P, indicative of reduced electron density around nitrogen (Figure 3c).^[^ ^44^ ^]^ A decrease in electron density enhances electrostatic attraction between the nucleus and electrons, increasing core‐electron binding energies. This can be associated with strong hydrogen bonding or ion‐pair interactions between ammonium ligands and surface halide ions (Figure S13), depleting electron density around nitrogen.

Fourier transform infrared (FTIR) spectroscopy further corroborates ligand modification by olefin metathesis. Compared to PNC‐P, PNC‐M exhibits increased intensity in the N–H bending or COO^−^ stretching (1580 cm^−1^), C–O stretching (1262 and 1085 cm^−1^), C–N stretching (1030 cm^−1^), and N–H wagging (806 cm^−1^) (Figures 3d and S14).^[^ ^45^, ^46^, ^47^, ^48^ ^]^ The apparent enhancement of vibrational features associated with ligand binding groups is likely a result of the relative attenuation of long‐chain alkyl C–H stretching modes (Figure S14). These spectral changes suggest that olefin metathesis reduces ligand chain‐length while preserving the binding groups. This conclusion was reinforced by zeta‐potential measurements showing no significant changes in surface charge of PNCs (Figure 3e).

### Enhanced Colloidal Stability of Metathesized PNCs

**Figure 4:**
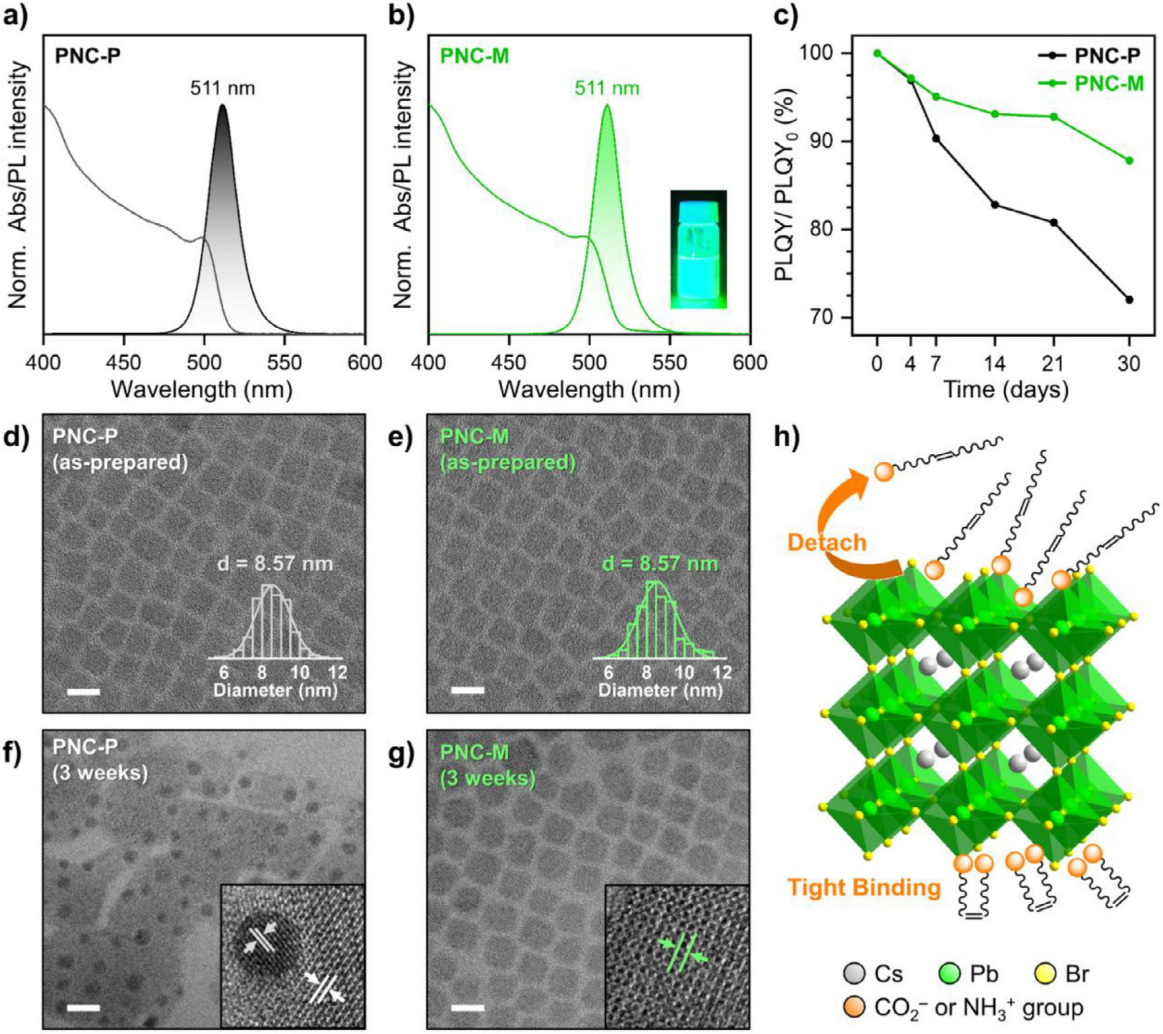
Characterization of PNCs with modified ligands. Absorption and emission properties of a) PNC‐P and b) PNC‐M (inset: photograph of PNC‐M solution under UV irradiation). c) PLQY changes of PNC‐P and PNC‐M over time under ambient conditions. d–g) TEM images of d,e) as‐prepared PNCs and f,g) PNCs after 3 weeks under ambient conditions. Scale bar: 10 nm. h) Illustration showing PNCs passivated with easily detached OA/OLAM ligands (above) and tightly bound metathesized ligands (below).

The impact of olefin ligand metathesis on the optical properties and colloidal stability of PNCs was systematically investigated. First, we verified that metathesis reactant does not alter the electronic structure or intrinsic optical properties of PNCs (Figure S15). Absorption and photoluminescence (PL) spectra before and after the reaction showed that both PNC‐P and PNC‐M maintained identical optical characteristics (Figures 4a,b and S16). However, a significant difference in long‐term PLQY stability was observed. After three weeks under ambient conditions, PNC‐P showed a 20% reduction in PLQY from its initial value, whereas PNC‐M retained over 93% PLQY (Figure 4c). This indicates that metathesized ligands enhance surface passivation by effectively preventing ligand detachment and mitigating defect formation. Subsequently, to evaluate the structural stability, high‐resolution transmission electron microscopy (HR‐TEM) and powder X‐ray diffraction (XRD) analyses were conducted. Both PNC‐P and PNC‐M showed monodisperse NCs with an average size of 8.57 nm with cubic phase (Figures 4d,e and S17), confirming that olefin ligand metathesis does not affect the crystal structure of PNCs. However, after three weeks of air exposure, clear differences were observed in PLQY retention and structural integrity. In three weeks, PNC‐P exhibited a significant reduction in PLQY (∼20%), which is attributed to ligand detachment, leading to surface degradation and metallic Pb formation (Figure 4c,f). HR‐TEM analysis also showed the presence of Moiré fringes in PNC‐P (Figure S18), indicating lattice distortion and partial phase decomposition. In contrast, PNC‐M showed only a 7% PLQY decrease under the same conditions, demonstrating that the metathesized ligands enhance the binding affinity of surface ligands to the PNC surface and effectively preserve structural integrity (Figure 4c,g). The removal of long hydrocarbon chains enhances chelating interactions with the PNC surface, thereby suppressing ligand detachment and maintaining colloidal stability. Taken together, these results indicate that the weakly bound native ligands in PNC‐P detach easily, leading to structural degradation, whereas the metathesized ligands in PNC‐M exhibit stronger surface binding, thereby ensuring prolonged colloidal stability (Figure 4h).

**Figure 5:**
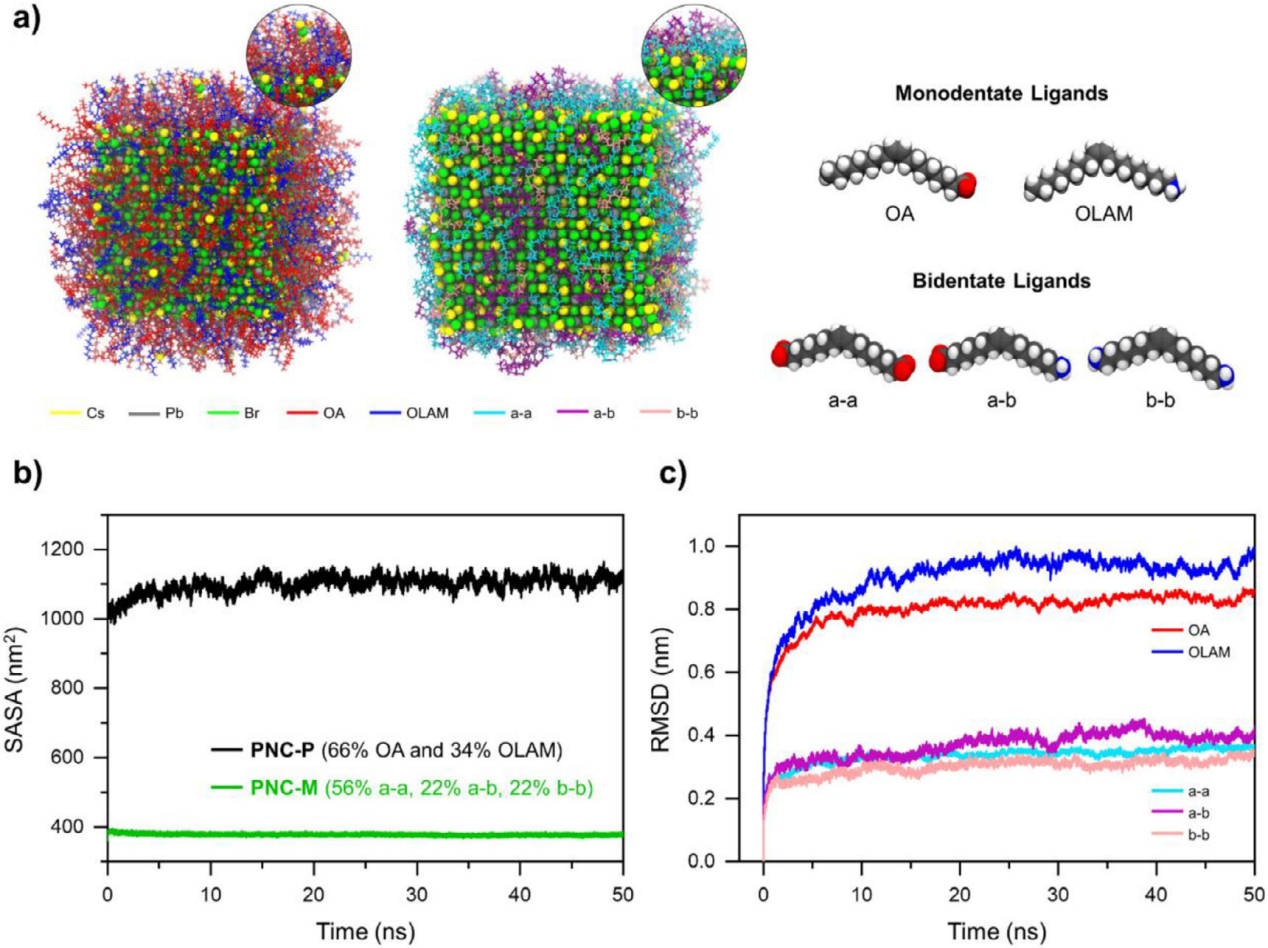
a) Models of PNC‐P and PNC‐M, along with their respective ligand types. Plots of the b) SASA analysis and c) RMSD. The OA and OLAM ligands correspond to PNC‐P, while the a‐a, a‐b, and b‐b ligands correspond to PNC‐M.

To reveal the atomistic origin underlying the superior performance of PNC‐M over PNC‐P, we conducted classical force field calculations for both systems, using a force‐field parameter set for the CsPbBr_3_ capped with various anchoring groups developed by some of us.^[^ ^49^ ^]^ The pristine PNC‐P was modeled with a 2:1 OA:OLAM ratio, aligned with the experimental conditions, incorporating 360 OA ligands (60% surface coverage) alongside 180 OLAM ligands (31.5% surface coverage) (Figure 5a). The corresponding bidentate‐ligand‐capped system, PNC‐M, was constructed with three distinct bidentate ligands: octadec‐9‐enedioate (a‐a), 17‐ammonioheptadec‐9‐enoate (a‐b), and hexadec‐8‐ene‐1,16‐diaminium (b‐b). In this nomenclature, “a” signifies the carboxyl (COO^−^) group that anchors to the PNC, while “b” denotes the ammonium (NH_3_^+^) anchoring group. For this bidentate system, we distributed 150 molecules of the a‐a type and 60 molecules each of the a‐b and b‐b types. Overall, the number of anchoring groups of COO^−^ and NH_3_^+^ types was kept constant between PNC‐P and PNC‐M, ensuring consistency in identifying eventual discrepancies in their behavior.

On both PNC‐M and PNC‐P, we performed a 50‐ns production run under isothermal‐isobaric (NPT) ensemble conditions at 1 atm and 300 K (see more details in the Supporting Information Texts S2, S3; Figure S19). We then analyzed their trajectories to analyze the stability of the capping layers. At first, we computed the solvent‐accessible surface area (SASA) of the capping ligands, a parameter that quantifies how much the ligand surface is accessible to solvent molecules. Analysis of the SASA plots revealed clear distinctions between PNC‐P and PNC‐M (Figure 5b). PNC‐P exhibited the higher and significantly more fluctuating SASA values, indicating weaker ligand packing and enhanced solvent‐ligand interactions, which may facilitate ligand desorption. In contrast, PNC‐M exhibited consistently lower and less fluctuating SASA values, indicating greater structural compactness and reduced solvent accessibility compared to the PNC‐P capped with OA/OLAM ligands. The bidentate ligands in PNC‐M, firmly anchored to the PNC surface, confer superior structural stability and enhanced surface coverage compared to monodentate and flexible OA/OLAM ligands (Figure 5c).

To further substantiate these observations, we conducted a Root Mean Square Deviation (RMSD) analysis using GROMACS. RMSD is a quantitative measure that calculates the average deviation of atomic positions from a reference structure, usually taken as the last or initial frame in the production run, thus providing insight into structural stability and conformational flexibility during the simulation. The obtained results indicate that PNC‐M, capped with bidentate ligands, exhibited notably lower RMSD values, reflecting minimal deviation from their reference configurations and thus a superior binding to the PNC surface (Figure 5c). In contrast, monodentate ligand‐capped PNC‐P demonstrated higher RMSD values, suggesting greater structural flexibility and less stability, more prone to detachment, relative to their initial configurations (Figure 5c).

In addition to the SASA and RMSD analyses, we conducted a radius of gyration (R_g_) analysis. Like SASA and RMSD, the R_g_ provides a measure of the overall size and structural compactness of a molecular system by quantifying the mass distribution relative to its center of mass. Our results reveal that the PNC‐M exhibits a notably lower and more stable R_g_, indicating enhanced compactness and structural stability compared to the PNC‐P (Figure S20). The structural advantages of bidentate ligand passivation persisted upon varying the composition of the monodentate ligand system, as demonstrated by additional simulations employing a representative 1:1 of OA:OLAM ratio (Supporting Information Texts S2, S3; Figure S21). Particularly, the SASA analysis suggests that increasing the proportion of the bidentate ligand type a–a further enhances PNC stability (Figures 5b and S21a), likely due to stronger chelation arising from the simultaneous coordination of two oleate groups to Pb^2+^ ions at the PNC surface. These findings support the robustness of bidentate anchoring, independent of the monodentate ligand composition.

Furthermore, we investigated whether the improvements observed in PNC‐M could also be achieved during direct synthesis without relying on olefin ligand metathesis. In particular, we examined whether introducing short‐chain carboxylic acids with terminal C═C bonds or bidentate ligands containing alkene moieties at the synthesis stage could produce comparable effects. We synthesized PNCs using various ligand combinations (Figure S22; Table S3), including conventional monodentate ligands (OA and OLAM), shorter‐chain alternatives (9‐decenoic acid (9‐DA) and octylamine (8‐AM)), and a bidentate octadec‐9‐enedioic acid (a–a). The a–a ligand was synthesized via self‐metathesis of OA (Figures S23, S24) and selected based on MD simulations, which presented a reduction in SASA and improved colloidal stability upon its incorporation (Figures 5b and S21). Among the synthesized PNCs, only one sample exhibited improved PLQY, though this enhancement was accompanied by spectral broadening (Table S3). All other ligand combinations resulted in substantially lower PLQY, and samples with PLQY below 30% showed aggregation in TEM images (Figure S25). These findings highlight that olefin ligand metathesis provides an effective strategy for enhancing the long‐term stability of PNCs while preserving their optical performance, without the need for complex ligand design, synthesis, or stoichiometric optimization.

### Improved Photosensitivity of Metathesized PNCs

Finally, the improved photosensitivity of PNC‐M was demonstrated through enhanced performance in direct optical lithography. Since the concentration of PNCs significantly influences the lithography process,^[^ ^18^, ^19^, ^20^ ^]^ we initially equalized the absorbance of PNC‐P and PNC‐M to ensure uniform concentration (Supporting Information Text S4; Figure S26, S27).

**Figure 6:**
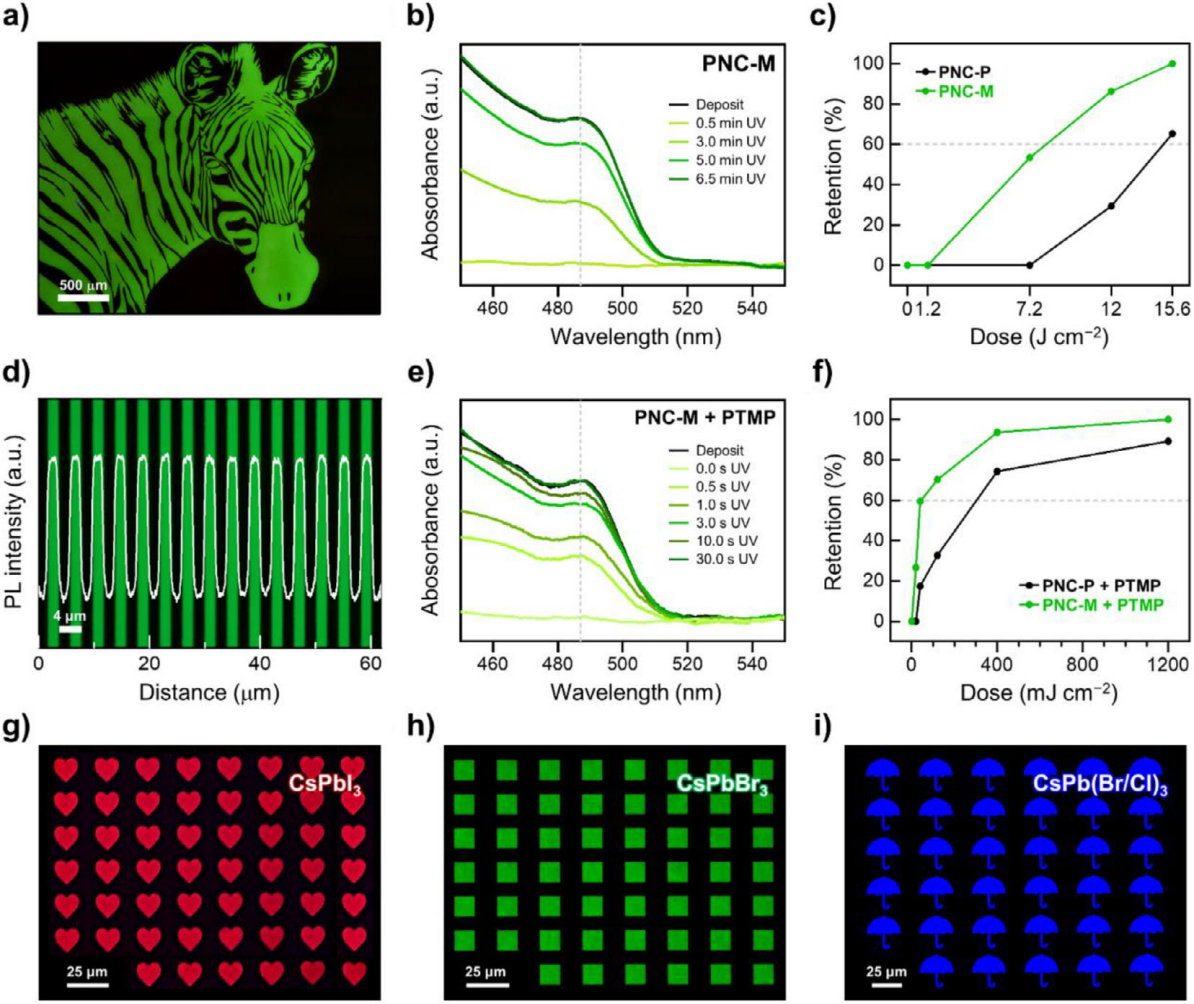
Direct optical lithography using PNCs via a–c) alkene polymerization and d–i) alkene hydrothiolation. a,d) Fluorescence OM image of zebra and 2‐µm line patterns from PNC‐M. d) PL intensity‐distance plot of 2‐µm line patterns overlaid with OM image. b,e) Absorption spectra of PNC‐M films after different UV exposure times. c), f) Film retention ratio of PNC‐P and PNC‐M as a function of UV dose. g–i) Multicolor patterning using CsPbX_3_ (X = I, Br, Br/Cl): red for CsPbI_3_, green for CsPbBr_3_, and blue for CsPb(Br/Cl)_3_.

Upon UV irradiation, photocatalytic radical formation occurs in PNCs. The generated radicals initiate photochemical reactions such as alkene polymerization^[^ ^50^, ^51^, ^52^, ^53^ ^]^ and hydrothiolation^[^ ^18^, ^54^ ^]^ within olefin ligands (Figure S28). These reactions facilitate the crosslinking between adjacent PNCs, altering their solubility. In PNC‐P, steric hindrance of the outer hydrocarbon chain inhibits alkene polymerization, requiring higher UV dose for patterning. In contrast, the exposed alkene moieties in PNC‐M enable easier crosslinking and efficient pattern formation. By utilizing the enhanced patternability, precise zebra, and line patterns were successfully formed in PNC‐M without any additives (Figures 6a and S29). To systematically determine the optimal UV (365 nm, 40 mW cm^−2^) dose for pattern formation, film retention ratio was evaluated by measuring film absorbance. For direct optical lithography via alkene polymerization, PNC‐M retained a complete film at a UV dose (365 nm, 15.6 J cm^−2^), whereas PNC‐P retained only 65% (Figure 6b,c). To achieve distinguishable patterns (retention>60%), PNC‐M required approximately half the UV dose (365 nm, 7.9 J cm^−2^) compared to PNC‐P (365 nm, 15.1 J cm^−2^) (Figure S30). The UV dose of 7.9 J cm^−2^ for PNC‐M is lower than previously reported values (365 nm, 18.0–194.4 J) for alkene polymerization in PNCs,^[^ ^52^, ^53^ ^]^ demonstrating the feasibility of patterning with reduced energy.

To further verify the enhanced photosensitivity of PNC‐M, alkene hydrothiolation‐based optical lithography was conducted using PTMP as thiol additive (Figure S28c). Initially, 10 wt% PTMP resulted in complete film retention at low UV dose (365 nm, 600 mJ cm^−2^), but this high concentration induced significant PNC aggregation.^[^ ^18^ ^]^ Therefore, the PTMP concentration was reduced to approximately 1.5 wt% to minimize the influence of PTMP and to evaluate the photosensitivity of PNCs accurately. Under these conditions, both PNC‐P and PNC‐M produced distinct patterns at low UV dose, but there is a notable difference in film retention ratio (Figures 6d–f and S31). The PTMP‐added PNC‐P exhibited a retained nearly 75% of film at 400 mJ cm^−2^, whereas PNC‐M completely retained the film under the same conditions. Specifically, PNC‐M requires only 40 mJ cm^−2^ UV dose for clear pattern formation, which is nearly seven times lower than PNC‐P (∼270 mJ cm^−2^), highlighting the enhanced photosensitivity achieved through olefin ligand metathesis. The morphology of patterns was analyzed using atomic force microscopy (AFM) and scanning electron microscopy (SEM) analysis. AFM images of 5‐µm line patterns showed uniformly defined structures with a 20‐nm thickness and low surface roughness (Figure S32). SEM images of line patterns showed that PNCs remained in the UV‐exposed regions, whereas only the substrate surface was observed in the unexposed regions after development (Figure S33). Furthermore, the emission colors of PNCs could be tuned by varying the halide composition (I, Br, and Br/Cl mixed for red, green, and blue emission, respectively) (Figure S34). The metathesized CsPbX_3_ with PTMP successfully formed precise multicolor patterns, demonstrating the versatility of olefin ligand metathesis across different PNC compositions (Figure 6g–i). To demonstrate the broad applicability of our strategy, olefin ligand metathesis was extended to both III–V (InP) and II–VI (CdSe) QD systems. The metathesized QDs consistently exhibited enhanced long‐term PLQY retention (Figure S35) and photopatternability (Figure S36). For example, metathesized InP QDs (InP─M) retained 91.6% of their initial PLQY after 30 days, compared to 86.7% for pristine InP QDs (InP─P). Moreover, InP─M achieved 60% film retention under a UV dose of 3.7 J cm^−2^, compared to 5.9 J cm^−2^ for InP‐P.

## Conclusion

We have developed a nondestructive ligand modification strategy based on olefin metathesis that simultaneously enhances the colloidal stability and photosensitivity of PNCs. By employing ethylene‐mediated Hoveyda–Grubbs catalysis, native oleic acid, and oleylamine ligands were transformed into metathesized ligands featuring dual anchoring groups and shortened alkyl chains. This structural rearrangement strengthens surface‐ligand interactions via enhanced chelation and reduced conformational entropy, effectively suppressing ligand desorption and preserving colloidal stability. These effects are supported by MD simulations, which confirm that the metathesized ligands enhance the structural robustness of PNCs. Moreover, the removal of sterically hindering hydrocarbon chains further exposes reactive alkene moieties, increasing photosensitivity and enabling efficient photochemical reactions. Consequently, the metathesized PNCs exhibit long‐term PLQY retention and structural stability under ambient conditions, as well as efficient photopatternability through alkene polymerization and hydrothiolation under reduced UV doses. This strategy provides a generalizable and additive‐free approach for tailoring the surface chemistry of a broad range of emissive nanocrystals (II–VI, III–V QDs, and PNCs), and offers a promising route toward high‐resolution photopatterning platforms for next‐generation optoelectronic applications such as microdisplays and photonic devices.

## Supporting Information

The authors have cited additional references within the Supporting Information.^[^ ^55^, ^56^, ^57^, ^58^, ^59^, ^60^, ^61^, ^62^, ^63^, ^64^, ^65^, ^66^, ^67^, ^68^, ^69^, ^70^, ^71^, ^72^, ^73^, ^74^, ^75^ ^]^

## Conflict of Interests

The authors declare no conflict of interest.

## Supporting information



Supporting Information

## Data Availability

The data that support the findings of this study are available from the corresponding author upon reasonable request.
