# Supplementary material for: Olefin Ligand Metathesis for Colloidal Emissive Nanocrystals with Enhanced Stability and Photosensitivity
Source: Angew Chem Int Ed Engl. 2025 Aug 29;64(42):e202514802. doi: 10.1002/anie.202514802 (PMC12518706; doi:10.1002/anie.202514802)
Supplement: Supplementary file 1 — Supporting Information [file ANIE-64-e202514802-s001.pdf]

SUPPORTING INFORMATION

Olefin Ligand Metathesis for Colloidal Emissive Nanocrystals with Enhanced Stability and Photosensitivity

Seongbeom Yeon,[a] Yoseph Kim,[b] Abdessamad El Adel,[c] Jaeyeong Ha,[a] Seongkyu Maeng,[a] Youngjo Kim,[b] Ivan Infante,*[c,d] and Himchan Cho*[a,e]

[a] Seongbeom Yeon; Jaeyeong Ha; Seongkyu Maeng; Himchan Cho
Department of Materials Science and Engineering
Korea Advanced Institute of Science and Technology (KAIST)
291 Daehak-ro, Yuseong-gu, Daejeon 34141, Republic of Korea
E-mail: himchan@kaist.ac.kr

[b] Yoseph Kim; Youngjo Kim
Department of Chemistry
Chungbuk National University
1 Chungdae-ro, Seowon-gu, Cheongju-si, Chungcheongbuk-do 28644, Republic of Korea

[c] Abdessamad El Adel; Ivan Infante
BCMaterials, Basque Center for Materials, Applications, and Nanostructures
UPV/EHU Science Park, Leioa, 48940 Spain
E-mail: ivan.infante@bcmaterials.net

[d] Ivan Infante
Ikerbasque Basque Foundation for Science, Bilbao, 48009 Spain

[e] Himchan Cho
Graduate School of Semiconductor Technology, School of Electrical Engineering
Korea Advanced Institute of Science and Technology (KAIST)
291 Daehak-ro, Yuseong-gu, Daejeon 34141, Republic of Korea

Table of Contents

- **Experimental Procedure**
- **Characterization Techniques**
- **Terminology Note**
- **Supplementary Texts S1–S4**
- **Supplementary Figures S1–S27**
- **Supplementary Tables S1, S2**
- **Reference**

Experimental Procedure

1. Materials

The red InP QD is offered by Samsung Advanced Institute of Technology. Cesium carbonate (Cs_2CO_3, 99.9%, Aldrich), lead chloride ($PbCl_2$, 98%, Aldrich), lead bromide ($PbBr_2$, >98%, Aldrich), lead iodide (PbI_2, 99%, Aldrich), zinc bromide ($ZnBr_2$, >98%, TCI), zinc iodide (ZnI_2, 99%, Aldrich), sodium thiocyanate (NaSCN, >98%, Aldrich), 1-octadecene (ODE, >91%, Aldrich), oleic acid (OA, 90%, Aldrich), oleylamine (OLAM, 70%, Aldrich), toluene (anhydrous, 99.8%, Aldrich), ethylene (99.999%, Special Gas Co.), Hoveyda-Grubbs catalyst M720 (HGC, 97%, Aldrich), acetonitrile (>99.5%, Aldrich), ethyl acetate (anhydrous, 99.8%, Aldrich), ethyl vinyl ether (99%, Aldrich), 1-decene (>99.0%, Aldrich), 9-decenoic acid (9-DA, >95%, Aldrich), octylamine (8-AM, 99%, Aldrich), pentaerythritol tetrakis(3-mercaptopropionate) (PTMP, >95%, Aldrich), toluene-d_8 (99 atom % D, Aldrich), methanol-d_4 (99 atom % D, Aldrich). Ethylene was purified by passing through a column packed with BASF catalyst R3–11G, activated carbon and 4 Å molecular sieves. All materials except for ethylene were used without further purification.

2. Preparation of cesium oleate precursor

To prepare cesium oleate, 652 mg (2.0 mmol) of Cs_2CO_3 and 3 mL of OA were added to a three-neck round-bottom flask containing 10 mL of ODE. The flask was connected to a condenser, sealed with a rubber septum, and equipped with a thermocouple for temperature monitoring. To remove residual moisture and oxygen, the mixture was placed under vacuum at 120 °C for 2 h. After degassing, the temperature was rapidly increased to 150 °C under N_2 conditions.

3. Synthesis of green-emitting PNCs

552 mg (1.5 mmol) of $PbBr_2$ and 676 mg (3.0 mmol) of $ZnBr_2$ are added in 40 mL of ODE in a three-neck round-bottom flask. Subsequently, 8 mL of OA and 8 mL of OLAM were added. The solution was degassed under vacuum at 120 °C for 1 h. Following this step, the flask was purged with N_2, and the temperature was rapidly increased to 180 °C. Finally, 5 mL of the prepared cesium oleate precursor was rapidly injected to produce $CsPbBr_3$ nanocrystals. The reaction was promptly quenched using an ice-cold water bath. The product was subjected to centrifugation at 8,000 rpm for 5 minutes, after which the resulting precipitate was collected and redispersed in toluene. Then, an equal volume of ethyl acetate was added for further purification, followed by centrifugation at 10,000 rpm for 10 min. Finally, the NCs were dispersed in toluene for use.

4. Synthesis of red-emitting PNCs

The $CsPbI_3$ PNCs were synthesized by following the article with some modifications.[1] 160 mg (0.5 mmol) of PbI_2 and 96 mg (0.3 mmol) of ZnI_2 is added in 10 mL of ODE in a three-neck round-bottom flask. Subsequently, 2 mL of OA and 2 mL of OLAM were added. The solution was degassed under vacuum at 120 °C for 1 h. Following this step, 1 mL of the prepared cesium oleate precursor was rapidly injected to produce $CsPbI_3$ nanocrystals. The reaction was promptly quenched using an ice-cold water bath. The product was subjected to centrifugation at 8,000 rpm for 5 minutes, after which the resulting precipitate was collected and redispersed in toluene.

Separately, a saturated NaSCN solution was prepared by dissolving NaSCN in 0.8 mL of DMF and subsequently diluting it with 1.2 mL of toluene. The 10 μL of NaSCN solution was added to 3 mL of a centrifuged $CsPbI_3$ PNC solution. To induce precipitation of the PNCs, 3 mL of methyl acetate was introduced into the mixture. For further purification, an equal volume of ethyl acetate was added,

followed by centrifugation at 10,000 rpm for 10 minutes. Finally, the purified NCs were dispersed in toluene for further use.

5. Synthesis of blue-emitting PNCs

278 mg (1.0 mmol) of $PbCl_2$ and 183 mg (0.5 mmol) of $PbBr_2$ is added in 20 mL of ODE in a three-neck round-bottom flask. Subsequently, 2 mL of OA and 2 mL of OLAM) were added. The solution was degassed under vacuum at 120 °C for 1 h. Following this step, the flask was purged with N_2, and the temperature was rapidly increased to 180 °C. Finally, 3 mL of the prepared cesium oleate precursor was rapidly injected to produce $CsPbCl_{3-x}Br_x$ nanocrystals. The reaction was promptly quenched using an ice-cold water bath. The product was subjected to centrifugation at 8,000 rpm for 5 minutes, after which the resulting precipitate was collected and redispersed in toluene. Then, an equal volume of ethyl acetate was added for further purification, followed by centrifugation at 10,000 rpm for 10 min. Finally, the NCs were dispersed in toluene for use.

6. Synthesis of CdSe/$Cd_{1-x}Zn_xSe_{1-y}S_y$ QDs

The CdSe/$Cd_{1-x}Zn_xSe_{1-y}S_y$ were synthesized by following the article with some modifications. 552 mg (0.4 mmol) of CdO, 338 mg (8 mmol) of $Zn(OA)_2$, and 10 mL of OA are added in 30 mL of ODE in a three-neck round-bottom flask. The solution was degassed under vacuum at 120 °C for 1 h. Separately, 1 mmol of Se and 23 mmol of S are added in 10 mL TOP to prepare $TOPSe_{1-y}S_y$ solution. The solution was stabilized at 300 °C under vacuum then, 2 mL of $TOPSe_{1-y}S_y$ was quickly injected into the other flask. Subsequently 4 mL of OA was added after 10 min reaction with vigorous stirring and waited for 4 min. The reaction was promptly quenched using an ice-cold water bath, and 20 mL of toluene was added to the flask at room temperature.

7. Procedures for the olefin metathesis of PNCs

Olefin metathesis of nanocrystals was conducted in a stainless-steel autoclave equipped with a stirring bar. For the reaction, PNCs were mixed with a stock solution containing 0.2 wt% HGC and placed inside the autoclave. Ethylene gas was then introduced to achieve a pressure of 1.0 MPa, and the reaction mixture was stirred at room temperature for 3 h. After reaction, any residual ethylene was carefully removed from the autoclave. The resulting PNCs were then purified with acetonitrile and redispersed in toluene.

8. Synthesis of octadec-9-enedioic acid

0.3 mol of OA was placed in a 250 mL three-neck round-bottom flask and purged with N_2 for 30 min. 0.3 mmol (0.1 mol%) of HGC was added, and the mixture was heated to 45 °C with stirring for 48 h under N_2 atmosphere. 20 mL of ethyl vinyl ether was added dropwise to quench the reaction, and the solvent was removed. The crude solid was recrystallized twice from hexane/ethyl acetate to produce octadec-9-enedioic acid.

^{1}H NMR (CD_3OD, 400 MHz, ppm) δ = 5.39 (2H, *m*, –CH=CH–), 2.28 (4H, *t, J* = 7.2 and 7.6 Hz, –CH_2CO_2H), 1.98 (4H, *m*), 1.60 (4H, *m*), 1.32 (16H, *m*).

^{13}C NMR (CD_3OD, 100 MHz, ppm) δ = 177.8 (*s*, CO2H), 131.6 (*s*, CH=CH), 35.1 (*s*), 33.7 (*s*), 30.8 (*s*), 30.4 (*s*), 30.3 (*s*), 30.2 (*s*), 26.2 (*s*).

Characterization Techniques

1. Optical characterization

The absorption spectra were measured with a UV–vis spectrophotometer (JASCO V-770, Tokyo, Japan). The absolute PLQY and PL emission spectra were measured by using a spectrofluorometer (JASCO FP-8550, Tokyo, Japan) equipped with an integrating sphere. Time-resolved photoluminescence (TRPL) measurements were performed using a Fluorolog-QM spectrometer (Horiba). The 360 nm laser source was used for excitation. Optical microscope (OM) images were obtained by using Olympus BS51M. Fluorescence optical microscope (FOM) images were obtained by using Nikon Eclipse 80i.

2. Transmission electron microscopy

High-resolution transmission electron microscopy (TEM) images were obtained with Tecnai G2 F30 S-Twin (FEI company, Hillsboro, Oregon, USA; KAIST Analysis Center for Research Advancement (KARA), Daejeon, Republic of Korea). Field emission TEM measurements were conducted using Talos F200X with Super X, which is 4 windowless SDD EDS system (Thermo Fisher Scientific Instrument, Waltham, MA, USA; KARA, Daejeon, Republic of Korea). TEM samples were prepared by drop-casting diluted PNCs dispersion on FCF300-Cu TEM grids.

3. X-ray photoelectron spectroscopy

X-ray photoelectron spectroscopy (XPS) spectra were measured using a Nexsa G2 (Thermo Fisher Scientific Instrument, Waltham, MA, USA; KARA, Daejeon, Republic of Korea). The XPS sample was prepared in a powder form for measurement through an additional centrifugation step. All data calibrated by C1s peak to 284.6 eV.

4. X-ray diffraction spectroscopy

X-ray diffraction (XRD) analysis was performed with a high-resolution powder X-ray diffractometer (SmartLab, RIGAKU, Tokyo, Japan; KARA, Daejeon, Republic of Korea) with Cu Kα radiation (λ = 1.54 Å).

5. Nuclear magnetic resonance spectroscopy

^{1}H-nuclear magnetic resonance (NMR) measurements were conducted by AVNEO400, AS500 (Bruker Biospin). 2D NMR measurements were conducted by an Avance Neo 600 NMR spectrometer (Bruker Biospin, Rheinstetten, Germany; KARA, Daejeon, Republic of Korea) equipped with a cryoprobe prodigy. The samples were dissolved in toluene-d_8.

6. Fourier transform infrared spectroscopy

Attenuated total reflectance-Fourier transform infrared (ATR-FTIR) measurements were carried out by a Nicolet iS50 (Thermo Fisher Scientific Instrument, Waltham, MA, USA; KARA, Daejeon, Republic of Korea) at the condition of 4000 to 400 cm^{-1} with the resolution of 4 cm^{-1}.

7. Zeta-potential measurement

Zeta-potential analysis was performed on a Zetasizer nano zs (Malvern Panalytical, Malvern, UK).

Terminology Note

In this study, we classify alkenes based on the position of the C=C bond within the molecular backbone, and we define the types of vinylic protons accordingly:

Terminal alkenes: Alkenes in which the C=C bond is located at the end of a carbon chain, typically featuring a $=CH_2$ moiety. Terminal alkenes possess both:

- Geminal vinylic protons: Two protons attached to the same sp^2-hybridized terminal carbon ($=CH_2$).
- Vicinal vinylic proton: A proton located on the adjacent internal sp^2 carbon (R–CH=CH_2).

Examples: ODE, 1-decene

Internal alkenes: Alkenes where the C=C bond is embedded within the carbon chain and substituted on both sides by alkyl groups. Internal alkenes contain only:

- Vicinal vinylic protons: Protons located on adjacent sp^2 carbons across the double bond (–CH=CH–).

Example: OA, OLAM

A vinylic proton is defined as a proton directly bonded to a sp^2-hybridized carbon of a C=C bond. Terminal alkene species exhibit both geminal and vicinal vinylic protons, whereas internal alkene species contain only vicinal vinylic protons**. In the context of this study, the term vicinal vinylic proton specifically refers to the proton located on the internal sp^2 carbon (–CH=) of a terminal alkene (R–CH=CH_2).**

Supplementary Text

Text S1. Effects of ODE on olefin ligand metathesis

The ^{1}H NMR spectrum of PNC-P exhibits the presence of 1-octadecene (ODE), which contains a C17=C18 double bond capable of participating in metathesis reactions. ODE can potentially produce hexacos-9-enoic acid (9-HCA, $C_{26}H_{52}O_2$) and hexacos-9-en-1-amine (9-HCAM, $C_{26}H_{53}N$) as ligand products. These plausible ligands can be produced via cross-metathesis of ODE with OA and OLAM, respectively (Figure S4). If they were present on the PNC surface, a noticeable increase in inter-dot distance would be expected due to their longer alkyl chains. However, TEM analysis shows that PNC-M exhibits an average inter-dot spacing comparable to PNC-P (Figure S5), indicating that such long-chain ligands are unlikely to be bound to the surface.

Moreover, XPS analysis of the head-to-tail atomic ratio (C/(N+O)) provides additional evidence. The incorporation of 9-HCA/9-HCAM would increase the C/(N+O) ratio due to their longer chain. However, a clear decrease in the C/(N+O) ratio is observed after metathesis (Figure 3b), further indicating that longer chain ligands are not present in PNC-M.

Collectively, these results demonstrate that although ODE contains an internal alkene moiety, its participation in the metathesis reaction is negligible. This is likely due to the strong dependence of metathesis efficiency on alkene position, chain length, and steric hindrance. When ODE, OA, and OLAM interact with the active ruthenium species (Ru=), they may form distinct intermediates A, B, and C, respectively (Figure S6). Among them, intermediate A, derived from ODE, forms an asymmetric metallacycle that is both sterically and electronically less stable[2-4] than the symmetric intermediates B and C. As a result, intermediate A exhibits lower reactivity and is more prone to decomposition or reverse reactions.

Text S2. Molecular Dynamics (MD) Simulation

To construct the PNC model, we began by preparing a $CsPbBr_3$ NC with an edge length of 5 nm, where the surface is terminated with the CsBr layer. Surface Cs cations were selectively removed from the inorganic core, resulting in a charge-balanced $Cs_{1030}Pb_{1000}Br_{3030}$ structure. Using the Compound Attachment Tool (CAT) software,[5] we prepared a total of four PNCs, comprising two pristine PNCs, capped with oleate (OA) and oleylammonium (OLAM) ligands, and two PNCs capped with bidentate ligands. The first pristine PNC was capped with 45% surface coverage of both OA and OLAM, with an equal number of ligands of both types (270 ligands of each). In contrast, the second pristine PNC was prepared with a 2:1 OA:OLAM ratio, incorporating 360 OA ligands (60% surface coverage) alongside 180 OLAM ligands (31.5% surface coverage). For the remaining bidentate PNCs, we employed three distinct bidentate ligands: octadec-9-enedioate (a-a), 17-ammonioheptadec-9-enoate (a-b), and hexadec-8-ene-1,16-diaminium (b-b). In this nomenclature, "a" signifies the carboxyl (COO^-) group that anchors to the NC, while "b" denotes the ammonium (NH_3^+) anchoring group. In the first bidentate-capped PNC, the PNC was uniformly capped with 90 molecules of each ligand type. Meanwhile, the second bidentate-capped PNC was modified with a different distribution: 150 molecules of the a-a type and 60 molecules each of the a-b and b-b types. Each PNC model was then placed in a 22 nm cubic simulation box using PACKMOL,[6] with 45332 toluene molecules added as the coarse-grained solvent for both systems. To perform the MD simulations, we initially carried out a 5 ns equilibration under canonical (NVT) ensemble conditions to stabilize all investigated systems (OA-OLAM capped and bidentate ligand capped) at a constant volume and temperature of 300 K, to mimic the room temperature. As previously described, all simulations were conducted using toluene as the solvent, represented by a coarse-grained model.[7] Following the NVT equilibration performed with a 2 fs time step, an additional equilibration step was conducted for 2 ns under isothermal-isobaric (NPT) ensemble conditions at 1 atm pressure and maintained at 300 K. Throughout both the NVT and NPT equilibration stages, position restraints were applied to the core atoms of the PNC to facilitate gradual equilibration of the overall system. Upon completion of these equilibration steps, the system was considered adequately equilibrated at the desired temperature and pressure, allowing the removal of position restraints and progression to the production phase. Finally, a 50 ns MD simulation was then performed for the production run. All simulations were carried out using GROMACS version 2023.15-12.[8-14] Force field (FF) parameters for the OA/OLAM-capped $CsPbBr_3$ perovskite were developed using the auto-FOX package, based on data from previous work.[15-17] For the MD simulations, we applied the smooth particle mesh Euler (SPME) method with beta-Euler splines.[18] Temperature control at 300 K was maintained using a velocity-rescaling thermostat[19] while pressure at 1 atm was regulated by the extended-ensemble Parrinello-Rahman barostat.[20] A 1 nm short-range cutoff was applied for both Lennard-Jones and Coulombic interactions. The force field parameters for the OA and OLAM ligand tails were obtained using MATCH,[21] a toolkit for automated assignment of CHARMM-based[22] atom types and force field parameters through comparison with a chemical fragment database.

Text S3. The Bidentate Ligands Capping Workflow

To accurately compare the stability of the two PNCs, it was necessary for them to have an identical number of anchoring groups (COO^- and NH_3^+). For this reason, we chose 90 molecules for each type of bidentate ligand, ensuring a consistent total number of anchoring groups. To achieve uniform ligand placement, we first introduced 270 dummy atoms representing the carboxylate anchoring sites. Subsequently, we generated an equal number of dummy atoms for the ammonium anchoring group by converting the closest available cationic atoms, thereby ensuring proximity between anchoring sites and bidentate ligands. To further guarantee the successful attachment of the second anchoring group of each ligand to the NC, we selected 90 anionic dummy atoms and deleted their nearest 90 dummy atoms of the same type. For ligands anchoring solely via ammonium groups (b-b type), cationic cesium (Cs) atoms were similarly selected and converted to dummy atoms. In the case of the hybrid ligand (a-b), vacancies were created strategically to accommodate the second anchoring group. Half of these hybrid ligands were anchored via the cationic group and the other half via the anionic group. Finally, we performed a 5 ns NVT molecular dynamics simulation in vacuum to relax and stabilize the ligand attachment.

Text S4. Adjustment of PNC Concentration for Precise Photosensitivity Comparison

Since the concentration of PNCs significantly influences the lithography process, we initially equalized the dry mass of PNC-P and PNC-M to ensure uniform concentration. Notably, variations in free ligand content can also affect the measured dry mass. To eliminate this variable, PNC-P was subjected to the same purification process as PNC-M. This control ensured that both PNC-P and PNC-M underwent equivalent purification steps, minimizing differences in free ligand contents. According to the Beer-Lambert Law, equal concentrations of PNCs should exhibit identical absorption spectra due to their proportional relationship. However, PNC-M showed higher absorbance than PNC-P (Figure S19), indicating a greater NC density in PNC-M. Since olefin ligand metathesis does not affect the PNC core, the higher absorbance of PNC-M means reduction of ligands weight due to the removal of outer hydrocarbon chain. Therefore, to precisely compare photosensitivity, we adjusted their absorbance instead of dry mass (Figure S20).

Supplementary Figure

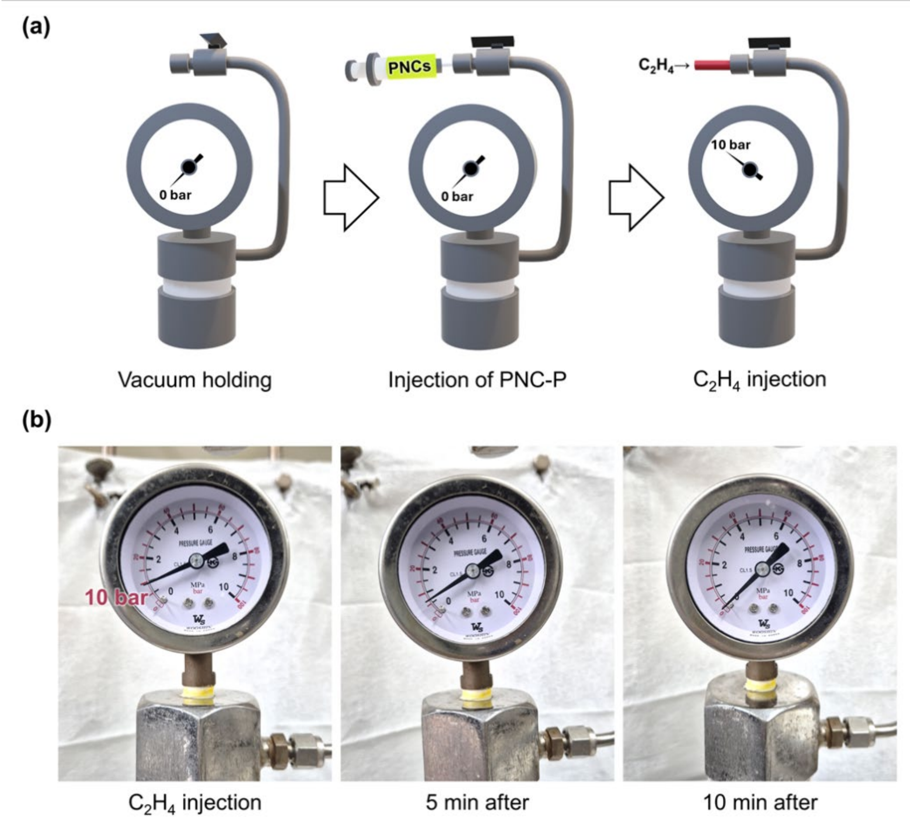

Figure S1. (a) Schematic illustration of C_2H_4 injection for olefin ligand metathesis. The process involves three steps: vacuum holding to remove air, injection of PNC-P under vacuum, and controlled injection of C_2H_4. (b) Changes in ethylene pressure. Initial pressure of 10 bar immediately after C_2H_4 injection, then reduction in pressure, indicating a reaction occurring within the system.

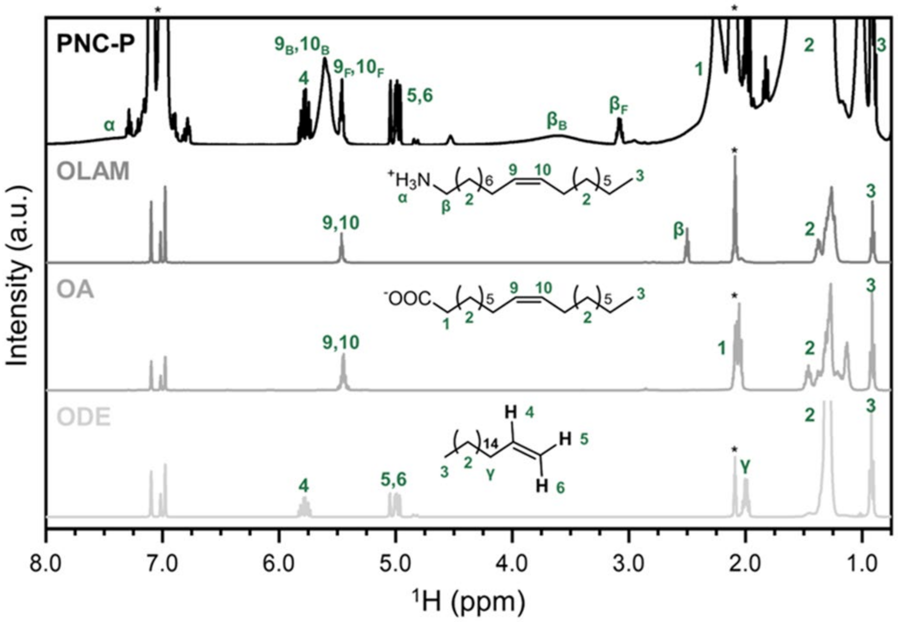

Figure S2. ^{1}H NMR spectra of PNC-P, OLAM, OA, and ODE. The asterisk symbol indicates toluene-d^{8}.

In the PNC-P spectrum, the characteristic peaks of OLAM and OA, corresponding to their aliphatic and unsaturated regions, are present with noticeable broadening and peak shifts. These changes suggest that the ligands coordinate with the PNC surface. Specifically, the α, β, and unsaturated (9,10) proton signals exhibit both broadening and chemical shift variations, which indicate both ligands remain attached to the PNCs, contributing to colloidal stability and surface passivation.

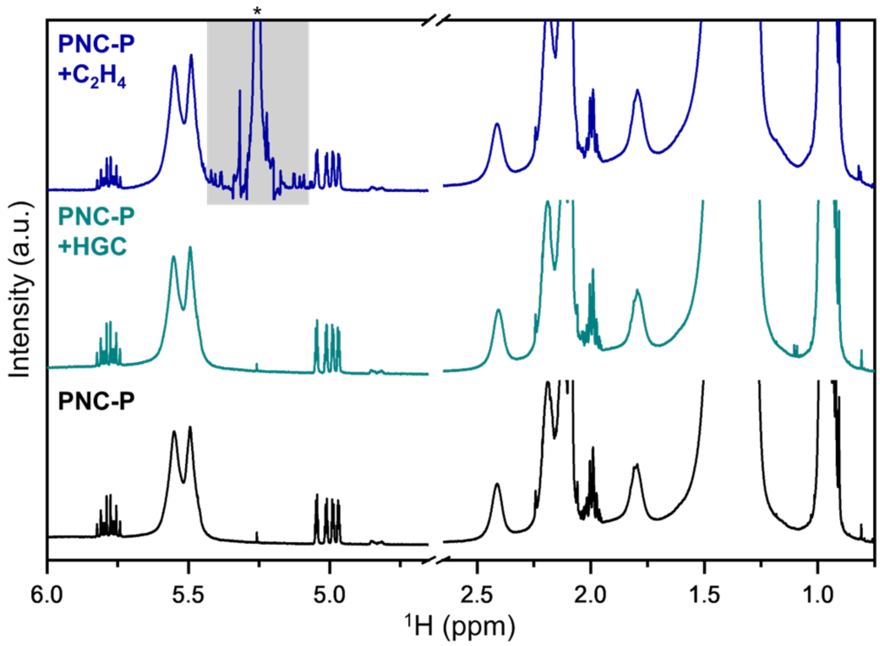

Figure S3. ^{1}H NMR of PNC-P before and after HGC and C_2H_4 addition, respectively. No spectral change is observed, indicating HGC and C_2H_4 do not affect the ligand environment of PNCs. The peaks in the gray region indicate ethylene.

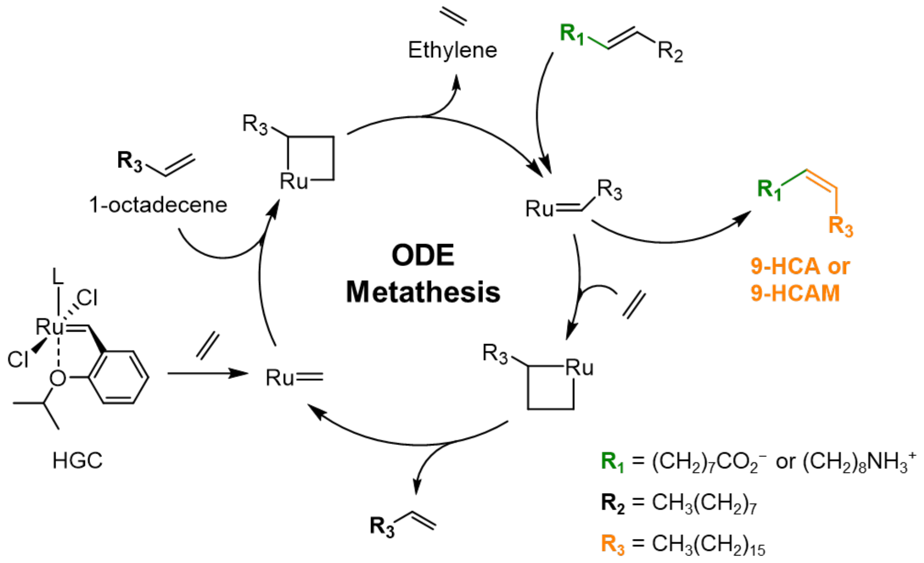

Figure S4. Proposed reaction mechanism of ODE-derived metathesis with native OA/OLAM ligands, resulting in the formation of 9-HCA or 9-HCAM.

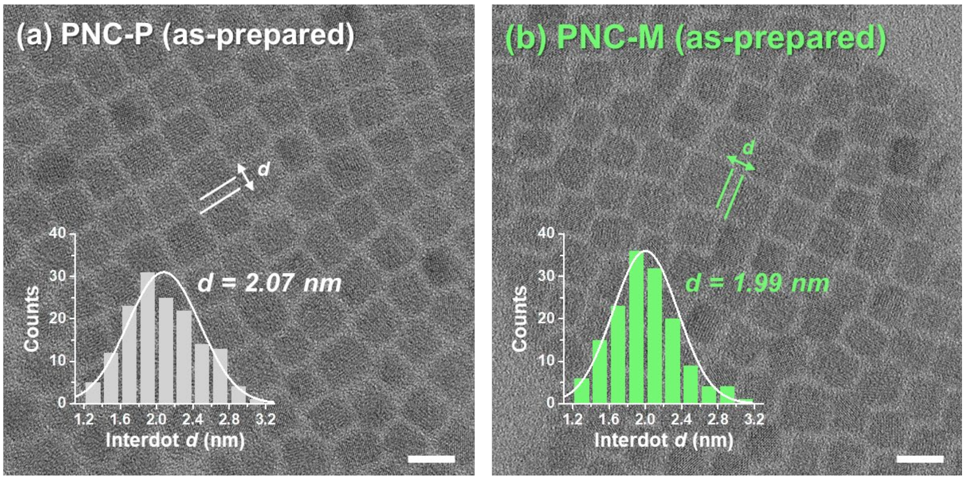

Figure S5. (a, b) Interdot distance of (a) PNC-P and (b) PNC-M. Scale bar: 10 nm.

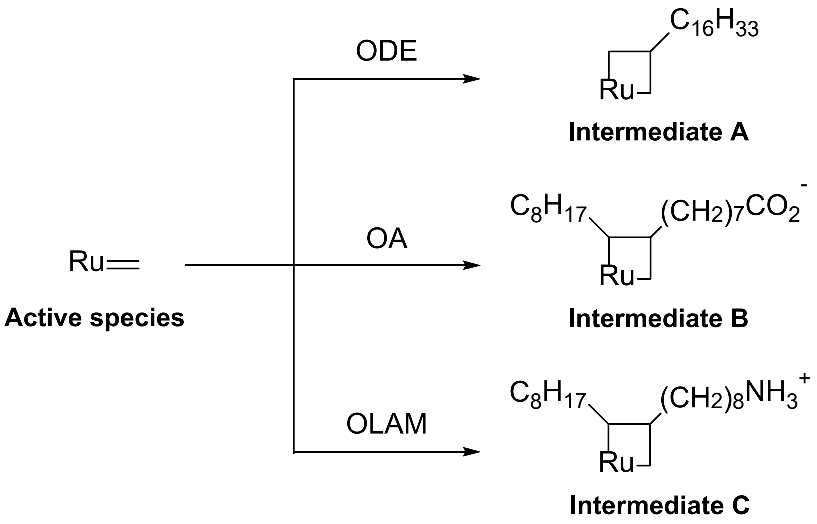

Figure S6. Metathesis of ODE, OA, and OLAM with the active ruthenium species (Ru=), forming intermediates A, B, and C, respectively.

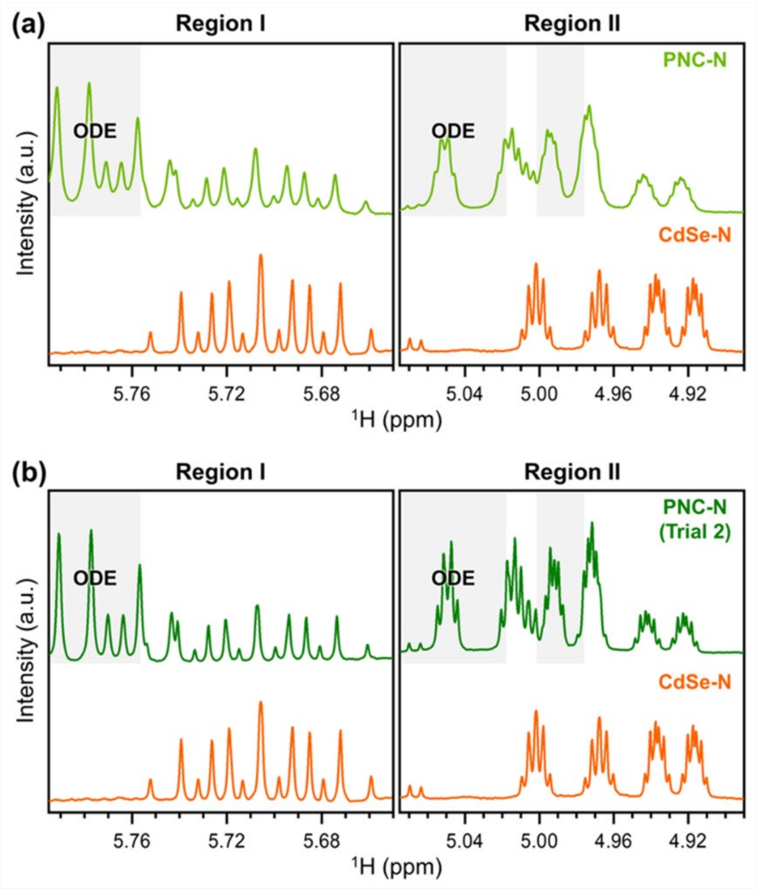

Figure S7. ^{1}H NMR spectra of terminal alkene in PNC-N and CdSe-N. (a) Region I (vicinal vinylic proton) and Region II (geminal vinylic proton) comparison between PNC-N and CdSe-N. (b) Reproducibility check with another PNC-N sample (Trial 2).

The identical peaks observed in PNC-N and CdSe-N suggest that the newly formed terminal alkene species result from ligand reactions. The gray-shaded areas indicate signals from ODE. Peak assignments for vicinal vinylic proton (Region I) and geminal vinylic proton (Region II) are described in **Table S1** and **Table S2**.

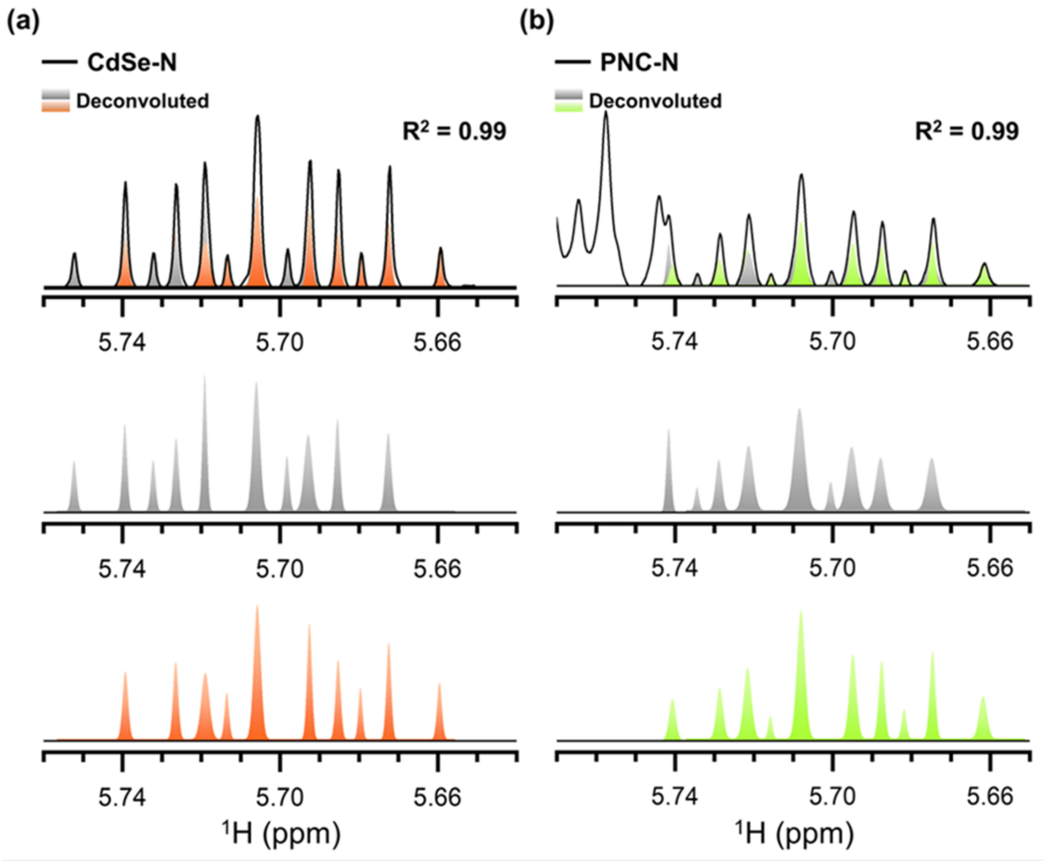

Figure S8. Deconvoluted ^{1}H NMR spectra of vicinal vinyl protons in (a) CdSe-N and (b) PNC-N. The deconvoluted peaks are overlaid with the original spectra, showing excellent fitting with R^2 = 0.99, confirming the consistency of peak assignments.

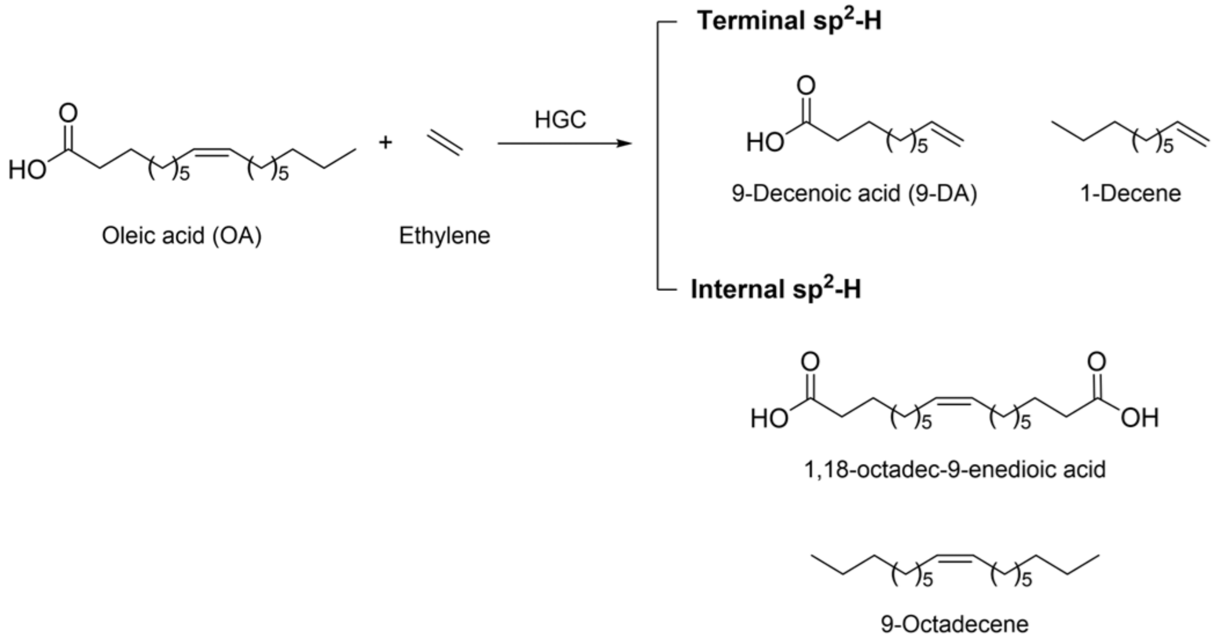

Figure S9. Reaction mechanism of the cross-metathesis between OA and ethylene, forming terminal and internal alkene species through catalytic C=C bond rearrangement.

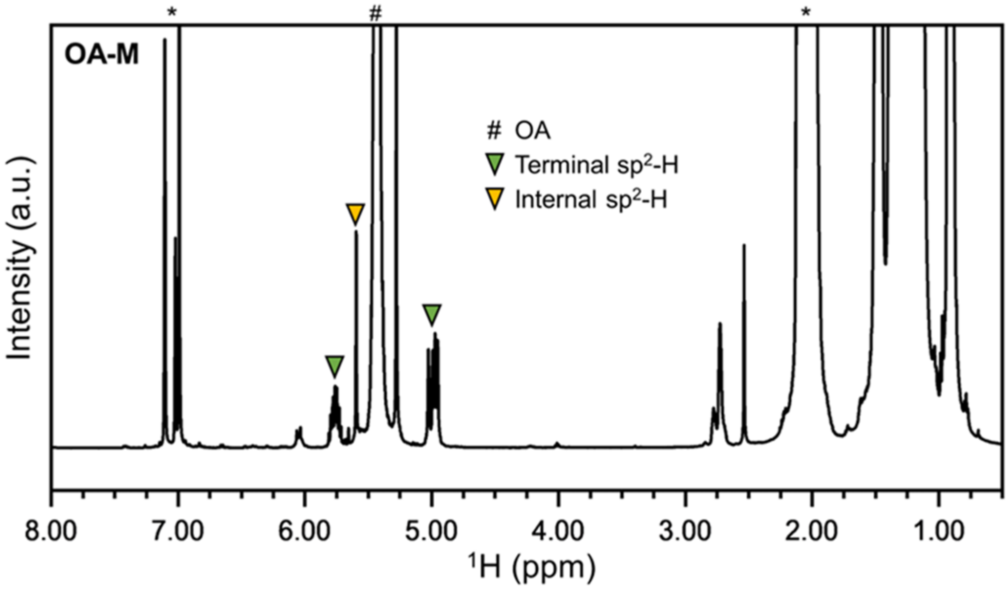

Figure S10. ^{1}H NMR spectrum of metathesized oleic acid (OA-M). The green and orange triangles indicate terminal and internal sp^2-H alkene species, respectively. The asterisk symbols indicate toluene-d^8.

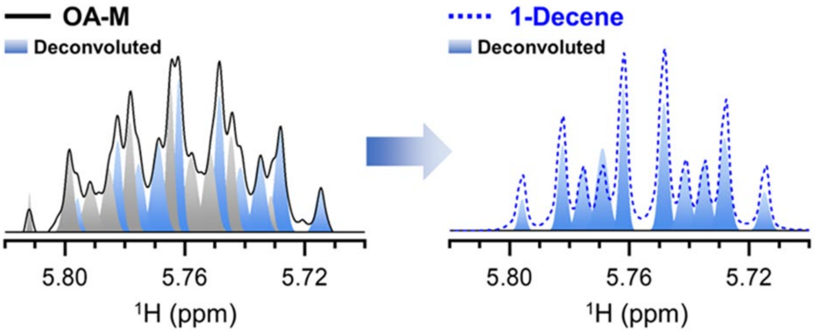

Figure S11. Deconvoluted ^{1}H NMR spectrum of OA-M for the vicinal vinylic proton (left). The blue deconvoluted peaks of OA-M exactly match the spectrum of 1-decene (right).

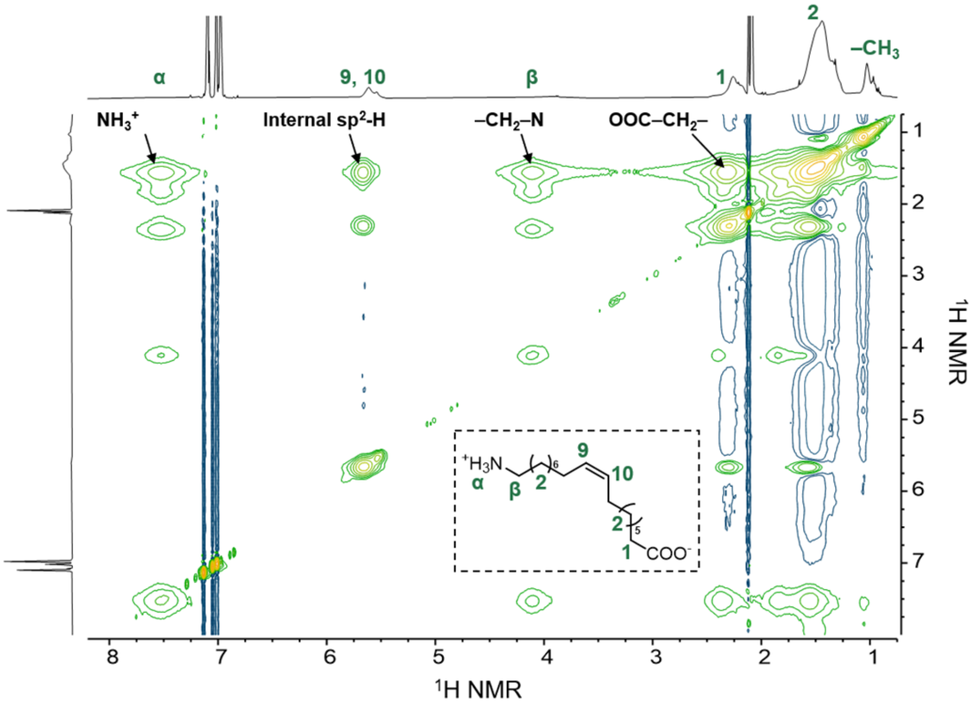

Figure S12. NOESY spectrum of PNC-M (inset: one of the plausible metathesized ligand molecules)

The Nuclear Overhauser Effect Spectroscopy (NOESY) provides information about through-space proton-proton interactions, allowing for the characterization of ligand environment and surface interactions. In the NOESY spectrum, diagonal peaks correspond to the direct ^{1}H chemical shifts, while off-diagonal cross-peaks indicate spatial proximity between specific protons, revealing their relative positioning within the system.

For PNC-M, the presence of a strong negative cross-peak suggests a ligand binding to the PNC surface. In contrast, the absence of cross-peaks between certain regions may indicate increased ligand mobility in solution, implying weaker interactions or partial ligand desorption. Specifically, in the NOESY spectrum of PNC-M, the strong negative peaks observed for NH_3, olefinic protons, $–CH_2–N$, and $OOC–CH_2–$ indicate that the metathesized ligands are tightly bound to the PNC surface. These interactions confirm the attachment of the modified ligands and suggest its significant role in stabilizing the PNC–ligand interface.

WILEY-VCH

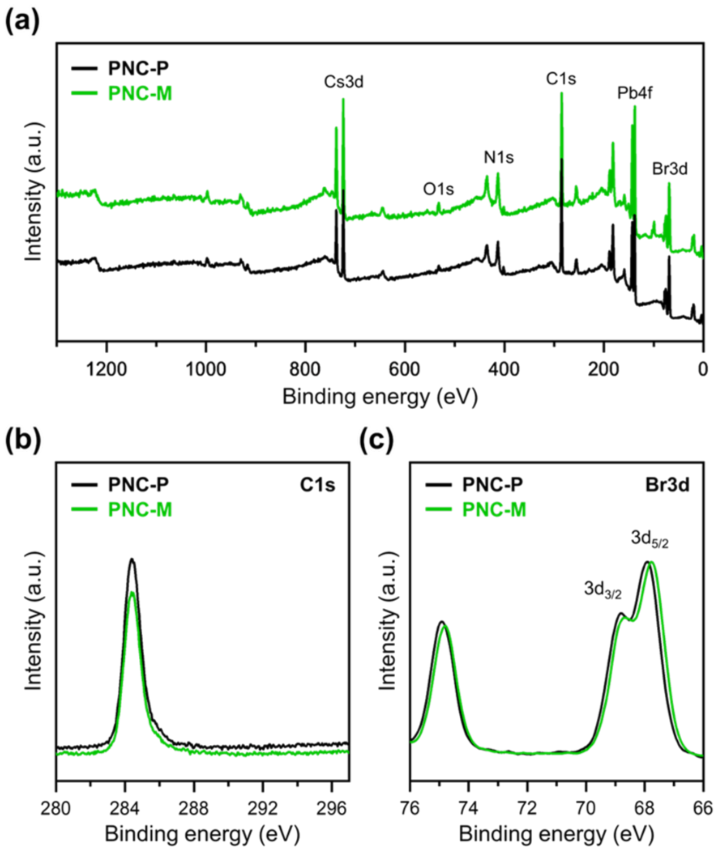

Figure S13. (a) XPS survey, (b) C1s, (c) Br3d spectra of PNC-P and PNC-M.

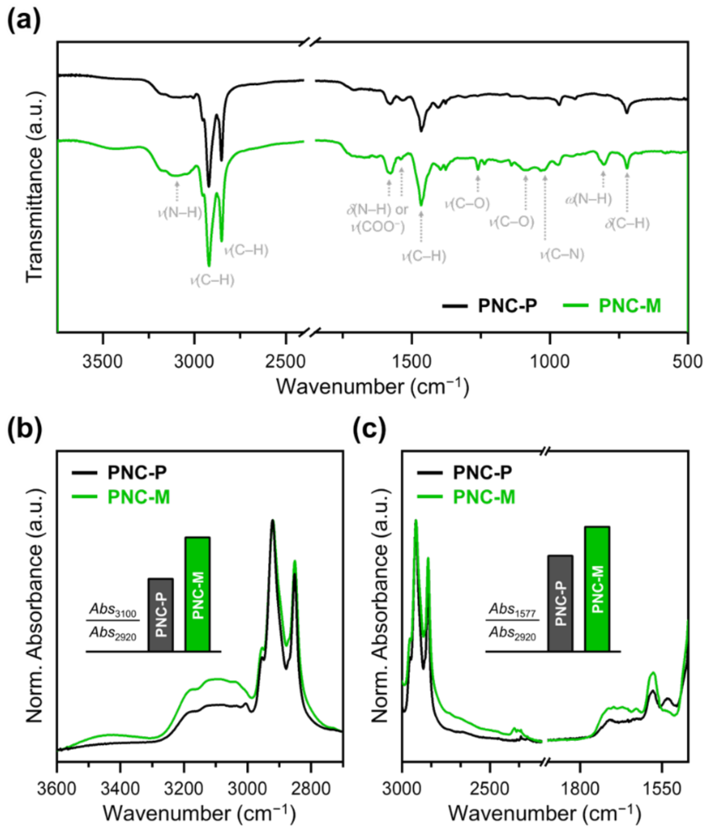

Figure S14. (a) FTIR spectra of PNC-P and PNC-M. (b) Ratio of *ν*(N–H) (3100 cm^{-1}) to *ν*(C–H) (2920 cm^{-1}). (c) Relative intensity of *ν*(COO^{-}) (1577 cm^{-1}) to *ν*(C–H). All spectra are normalized to the *ν*(C–H).

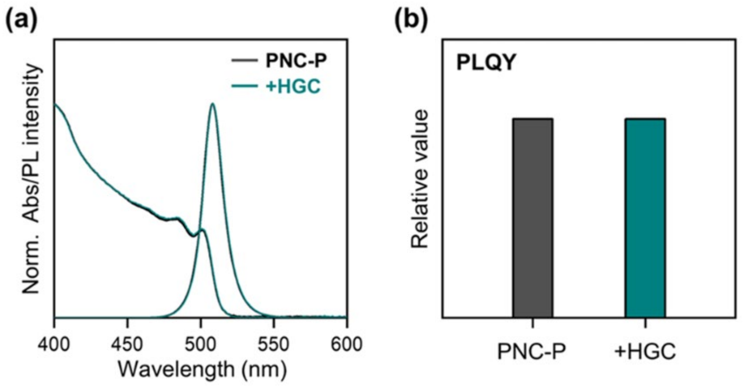

Figure S15. (a) Normalized absorption and PL spectra of PNCs before and after HGC addition. (b) Relative PLQY value of PNCs.

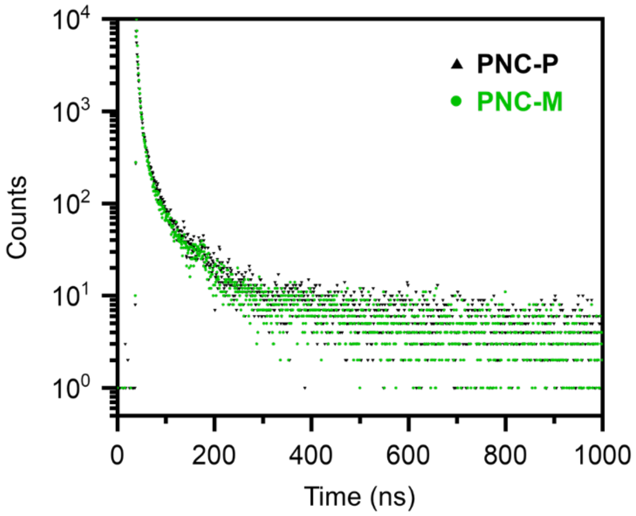

Figure S16. TRPL spectra of PNC-P and PNC-M.

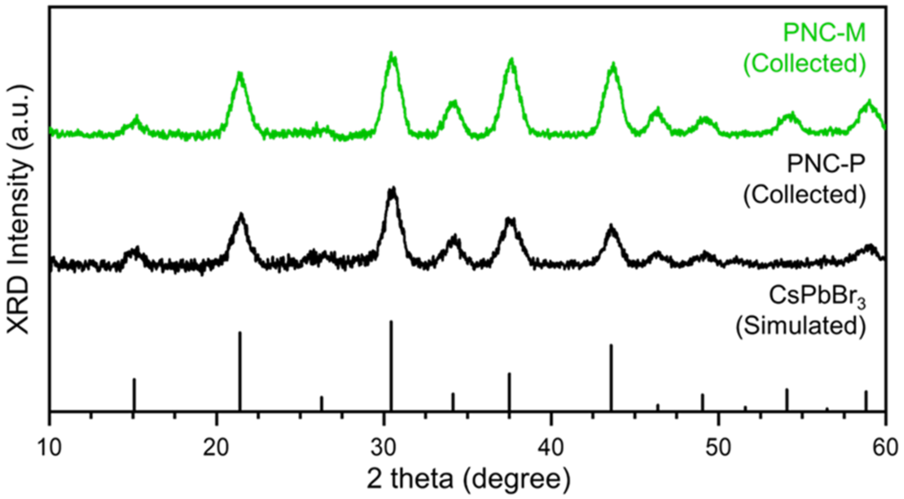

Figure S17. XRD patterns of PNC-P and PNC-M. The collected patterns are compared to simulated XRD patterns based on the ICSD database: $CsPbBr_3$ (ICSD 29073).

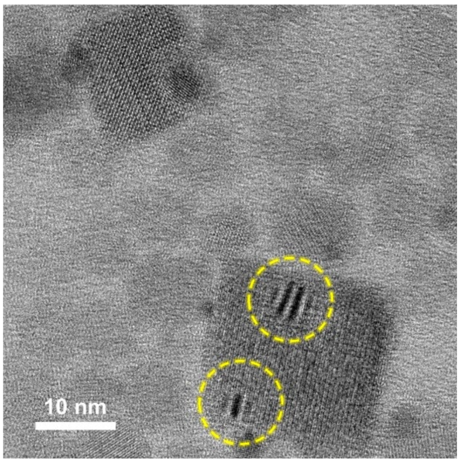

Figure S18. Observation of Moiré fringe in PNC-P.

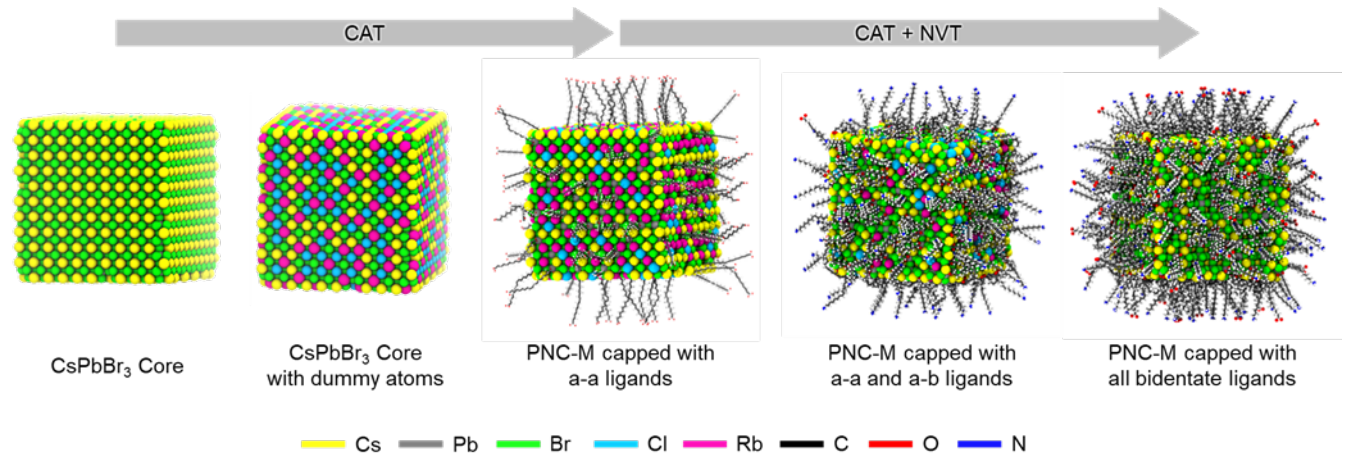

Figure S19. The steps to anchor the bidentate ligands.

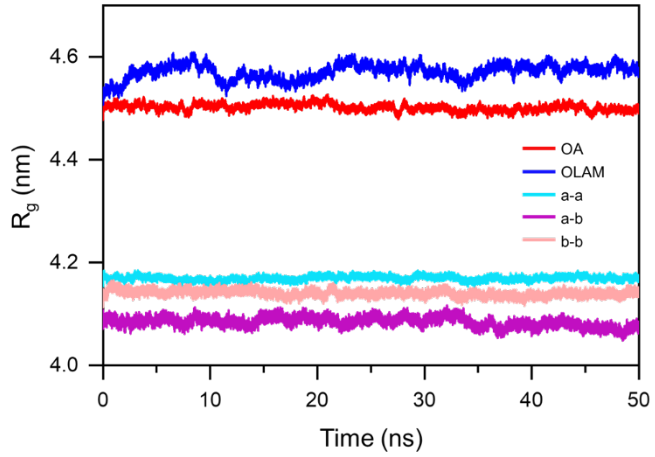

Figure S20. The radius of gyration (R_g) analysis. The OA and OLAM ligands correspond to PNC-P, while the a-a, a-b, and b-b ligands correspond to PNC-M.

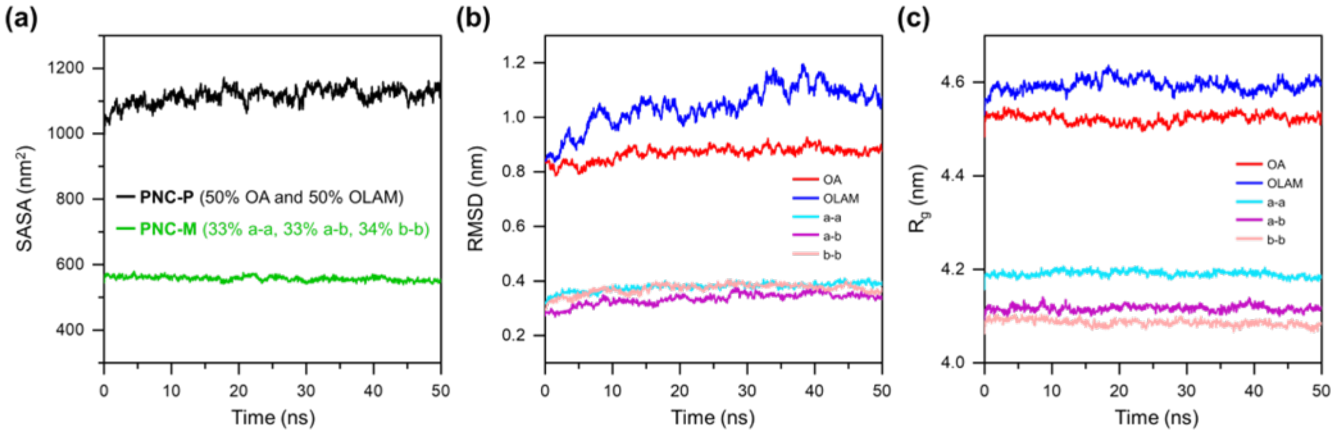

Figure S21. Plots of the (a) SASA analysis, (b) RMSD, and (c) R_g for PNC along with their respective ligand types. The OA and OLAM ligands correspond to PNC-P, while the a-a, a-b, and b-b ligands correspond to PNC-M.

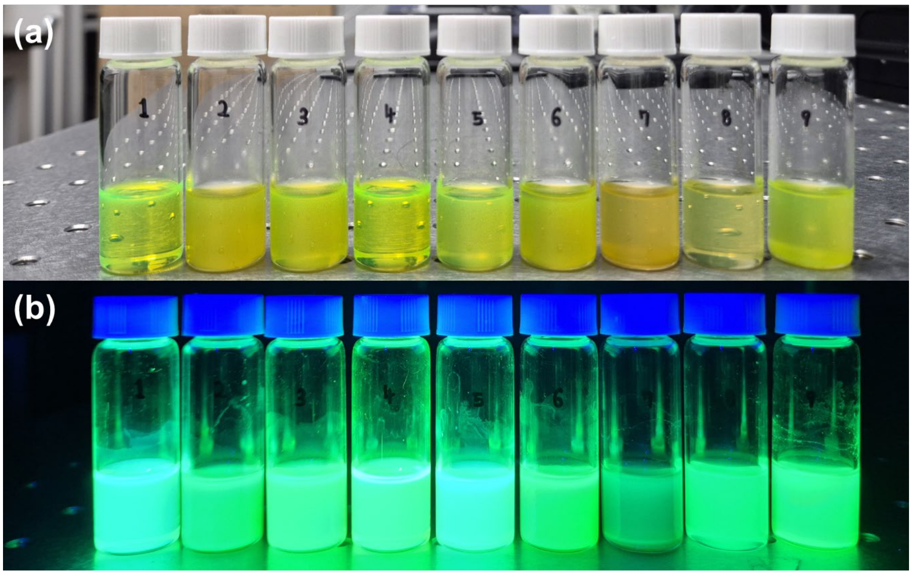

Figure S22. (a, b) Photographs of PNC sample 1–9, synthesized with various ligand combinations under (a) ambient light and (b) UV irradiation.

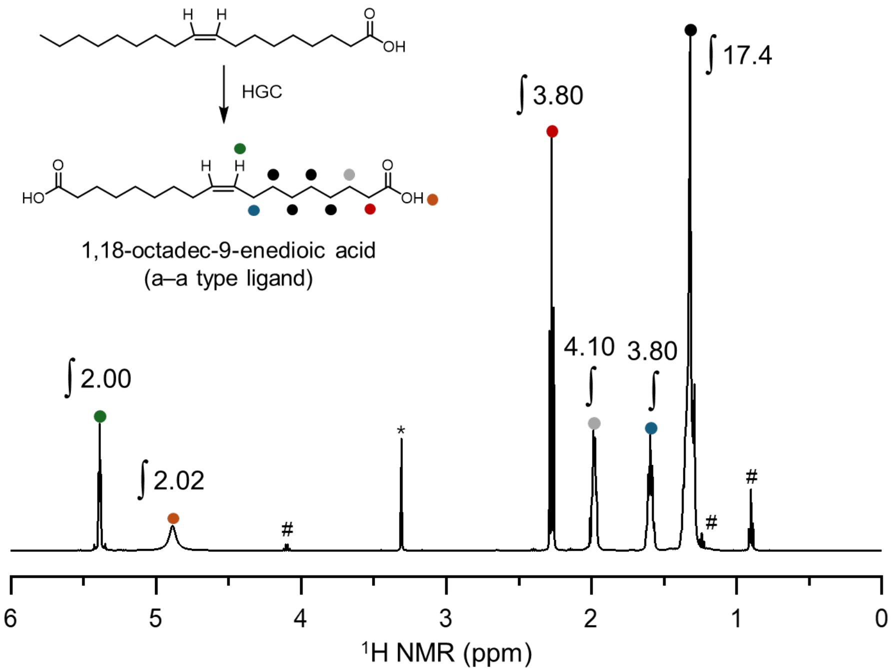

Figure S23. ^{1}H NMR spectrum of octadec-9-enedioic acid. The asterisk and hash symbols indicate methanol-d^4 and recrystallization solvents (hexane and ethyl acetate), respectively.

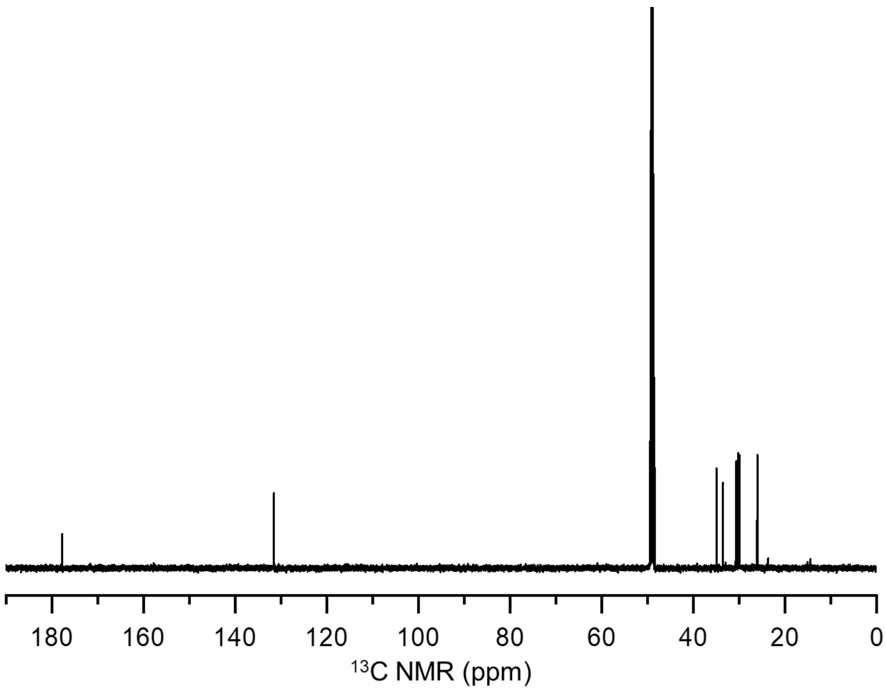

Figure S24. ^{13}C NMR spectrum of octadec-9-enedioic acid.

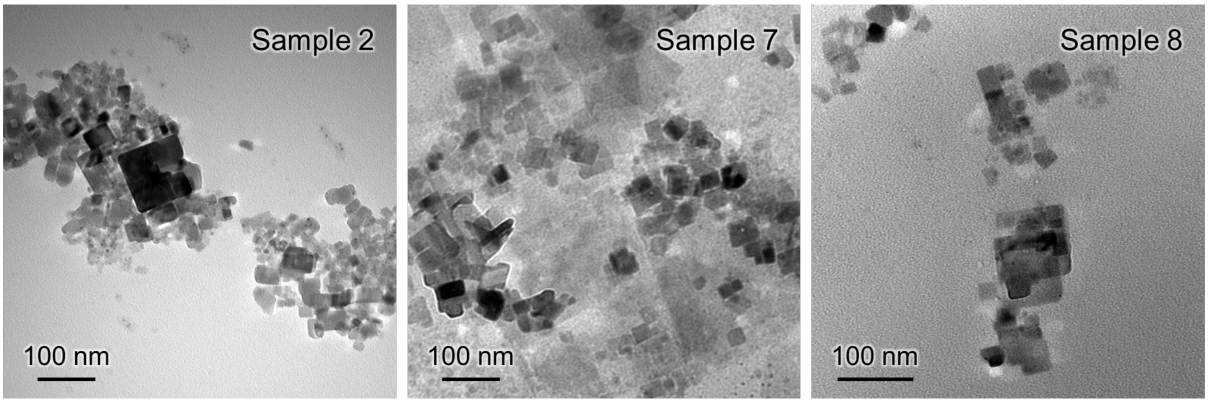

Figure S25. TEM images of sample 2, 7 and 8 in Figure S22 and Table S2.

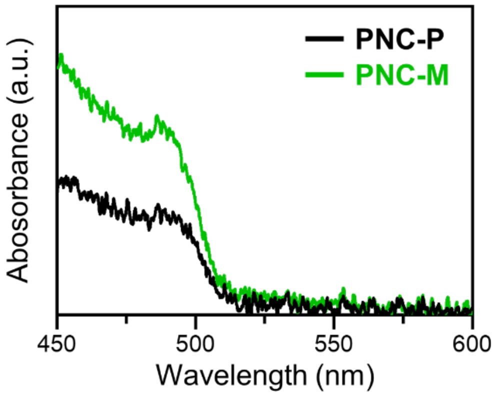

Figure S26. Absorption spectra of PNC-P and PNC-M at equal dry mass. PNC-M shows higher absorbance, indicating lower ligand molecular weight.

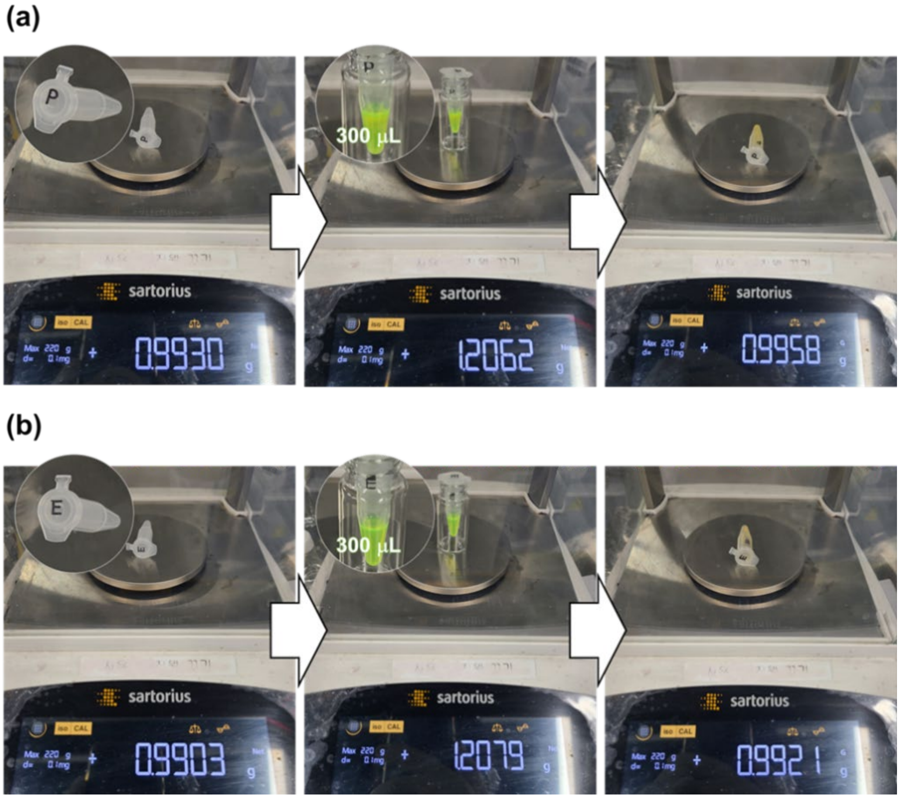

Figure S27. Dry mass measurements of PNCs at equal absorbance. (a) PNC-P (labelled as P) shows a dry mass of 9.33 mg mL^{-1}. (b) PNC-M (labelled as E) shows a smaller dry mass of 6.00 mg mL^{-1}, indicating lower ligand molecular weight.

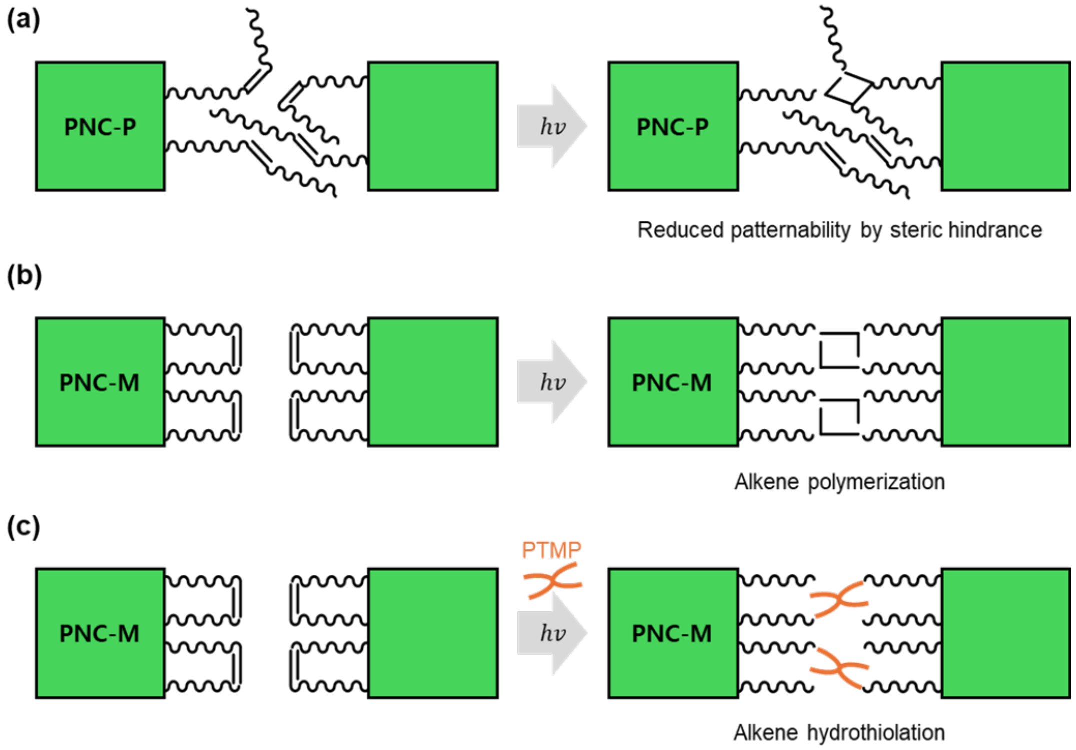

Figure S28. Schematic illustration of PNC crosslinking *via* alkene photochemical reactions. (a, b) Alkene polymerization of PNCs. (a) In PNC-P, steric hindrance from outer hydrocarbon chains prevents alkene polymerization. (b) In PNC-M, exposed alkenes enable efficient polymerization upon UV irradiation. (c) Alkene hydrothiolation-based crosslinking of PNC-M in the presence of PTMP.

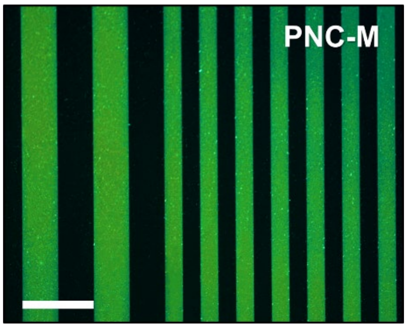

Figure S29. 50- and 25-nm line patterns produced using PNC-M. Scale bar: 100 nm.

WILEY-VCH

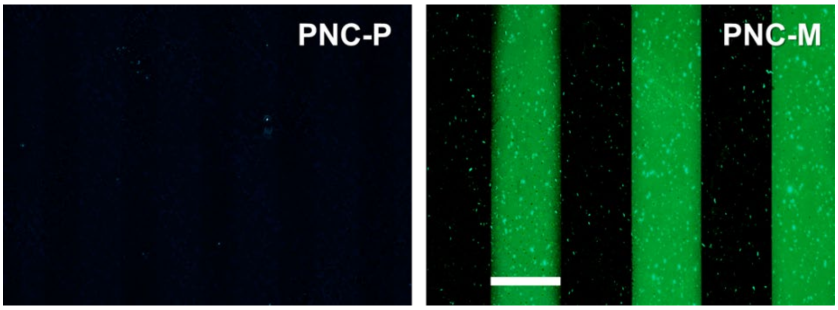

Figure S30. Patterning capability of PNCs upon exposure to 7.2 J cm^{-2} of 365 nm UV light. Distinct line patterns were observed for PNC-M, while PNC-P failed to form any patterns. Scale bar: 100 nm.

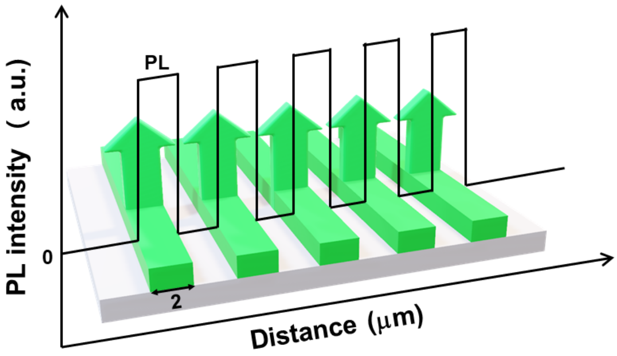

Figure S31. Detailed illustration for PL intensity-distance plot of 2-μm line patterns overlaid with OM image.

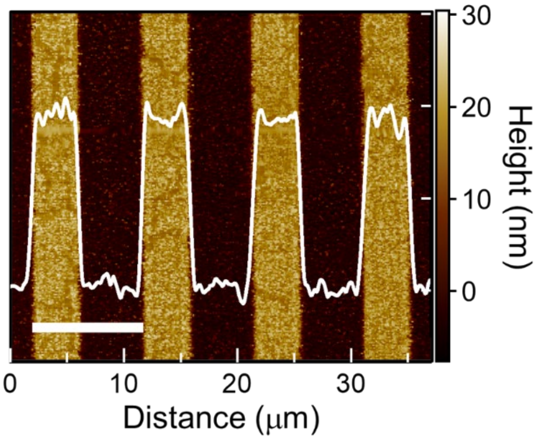

Figure S32. AFM images of line patterns formed with PNC-M. Pattern height–distance plot of a 5-μm line pattern overlaid with an AFM image. Scale bar: 10 μm.

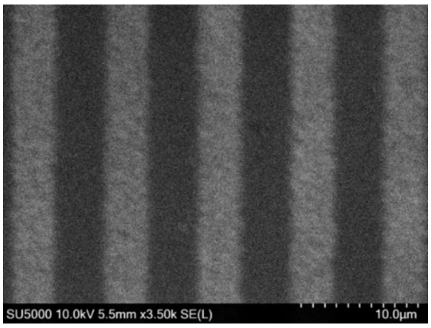

Figure S33. SEM image of line patterns produced using PNC-M.

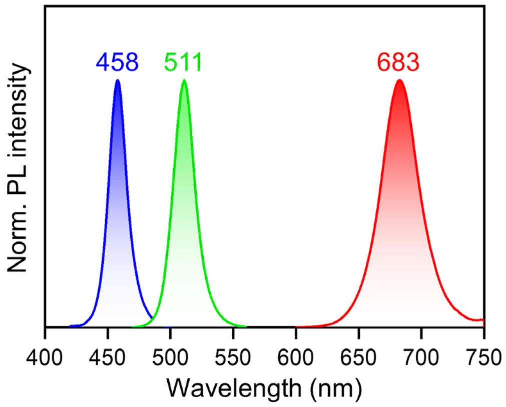

Figure S34. Emission wavelengths associated with the red, green, and blue luminescent patterns.

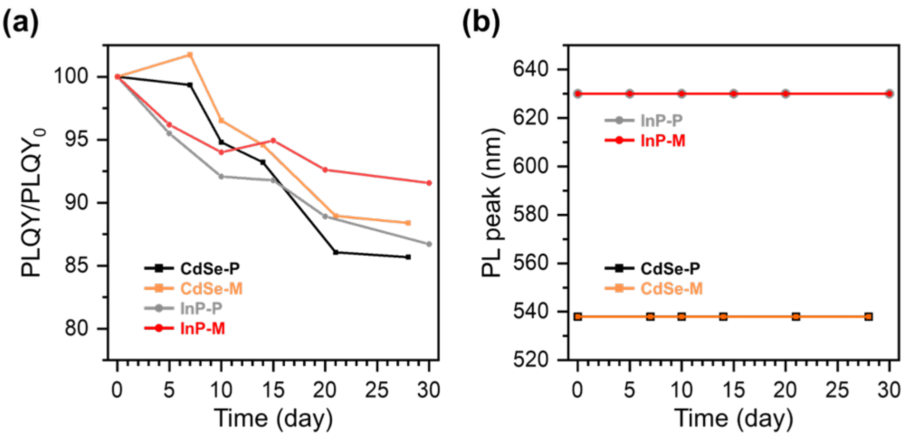

Figure S35. (a) PLQY changes and (b) PL peak position of pristine QDs (CdSe-P and InP-P) and metathesized QDs (CdSe-M and InP-M) over time under ambient conditions.

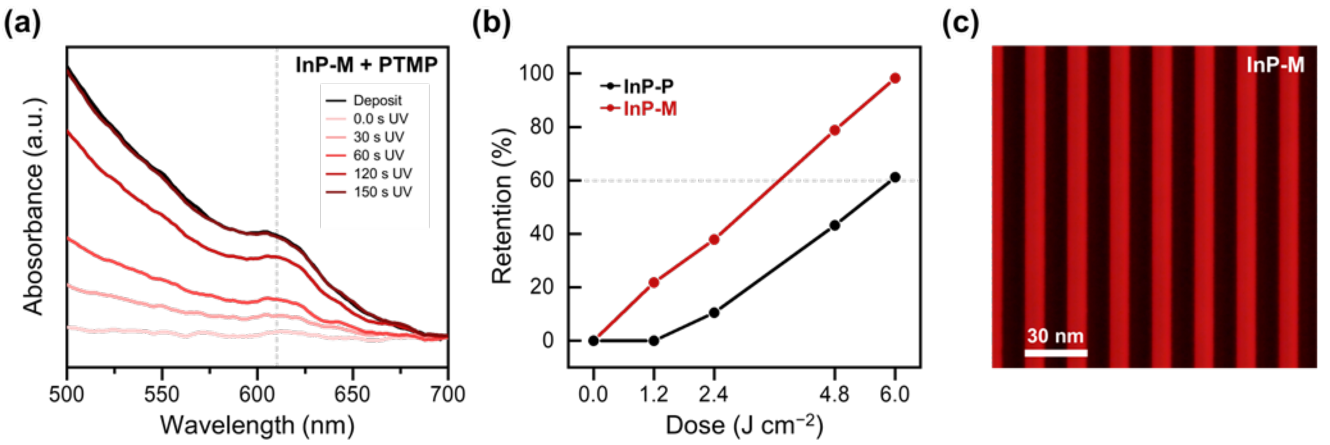

Figure S36. (a) Absorption spectra of InP-M films after different UV exposure times. (b) Film retention ratio of InP QDs as a function of UV dose. (c) Fluorescence OM image of 10-μm line patterns from InP-M. 5 wt% PTMP was added to the InP QD solution.

Supplementary Table

Table S1. Peak assignments of vicinal vinylic protons (Region I) as described in **Figure S7**.

PNC-N		CdSe-N		Delta (Hz)
^{1}H (ppm)	*J* (Hz)	^{1}H (ppm)	*J* (Hz)	
5.6608	-	5.6592	-	-
5.6737	6.45	5.6721	6.45	0
5.6809	3.6	5.6793	3.6	0
5.6866	2.85	5.6850	2.85	0
5.6939	3.65	5.6923	3.65	0
5.6996	2.85	5.6980	2.85	0
5.7072	3.8	5.7057	3.85	0.05
5.7149	3.85	5.7133	3.8	0.05
5.7206	2.85	5.7190	2.85	0
5.7278	3.6	5.7262	3.6	0
5.7336	2.9	5.7320	2.9	0

Table S2. Peak assignments of geminal vinylic protons (Region II) as described in **Figure S7**.

PNC-N		CdSe-N		Delta (Hz)
^{1}H (ppm)	*J* (Hz)	^{1}H (ppm)	*J* (Hz)	
4.9155	-	4.9101	-	-
4.9183	1.4	4.9129	1.4	0
4.9211	1.4	4.9157	1.4	0
4.9227	0.8	4.9173	0.8	0
4.9255	1.4	4.9201	1.4	0
4.9283	1.4	4.9229	1.4	0
4.9356	-	4.9303	-	-
4.9384	1.4	4.9330	1.35	0.05
4.9412	1.4	4.9358	1.4	0
4.9429	0.85	4.9375	0.85	0
4.9457	1.4	4.9402	1.35	0.05
4.9485	1.4	4.9430	1.4	0

Table S3. Emission properties of PNCs synthesized with various ligand combinations.

Sample	1	2	3	4	5	6	7	8	9
Ligands									
OA (mmol)	12.7	6.35	6.35	6.35	6.35	–	–	–	3.18
a-a (mmol)	–	3.18	3.18	–	1.59	3.18	3.18	–	6.35
9-DA (mmol)	–	–	–	6.35	3.17	6.35	6.35	12.7	–
OLAM (mmol)	12.3	12.3	6.15	6.15	6.15	6.15	–	–	12.3
8-AM (mmol)	–	–	6.15	6.15	6.15	6.15	12.3	12.3	–
Optical data									
λ_{em}(nm)	508	518	518	516	508	519	520	520	519
FWHM (nm)	18.9	22.2	18.7	17.4	24.4	18.5	19.5	18.4	18.8
PLQY (%)	90.7	18.9	42.3	73.6	94.5	26.8	11.8	29.6	74.4

Reference

[1] P. Lu, A. Liu, M. Lu, F. Zhang, S. Sun, M. Liu, Z. Wu, X. Wang, W. Dong, F. Qin, Y. Gao, X. Bai, Y. Zhang, Multi-Species Surface Reconstruction for High-Efficiency Perovskite Nanocrystal Light-Emitting Diodes, *Angew. Chem. Int. Edit.* **2024**, *63*, e202317376.

[2] K. Paredes-Gil, X. Solans-Monfort, L. Rodriguez-Santiago, M. Sodupe, P. Jaque, DFT study on the relative stabilities of substituted ruthenacyclobutane intermediates involved in olefin cross-metathesis reactions and their interconversion pathways, *Organometallics* **2014**, *33*, 6065-6075.

[3] A. Poater, L. Cavallo, A comprehensive study of olefin metathesis catalyzed by Ru-based catalysts, *Beilstein Journal of Organic Chemistry* **2015**, *11*, 1767-1780.

[4] O. Ogba, N. Warner, D. O'leary, R. Grubbs, Recent advances in ruthenium-based olefin metathesis, *Chemical Society Reviews* **2018**, *47*, 4510-4544.

[5] B. van Beek, J. Zito, L. Visscher, I. Infante, CAT: a compound attachment tool for the construction of composite chemical compounds, *Journal of chemical information and modeling* **2022**, *62*, 5525-5535.

[6] L. Martínez, R. Andrade, E. G. Birgin, J. M. Martínez, PACKMOL: A package for building initial configurations for molecular dynamics simulations, *Journal of computational chemistry* **2009**, *30*, 2157-2164.

[7] S. J. Marrink, H. J. Risselada, S. Yefimov, D. P. Tieleman, A. H. De Vries, The MARTINI force field: coarse grained model for biomolecular simulations, *The journal of physical chemistry B* **2007**, *111*, 7812-7824.

[8] M. J. Abraham, T. Murtola, R. Schulz, S. Páll, J. C. Smith, B. Hess, E. Lindahl, GROMACS: High performance molecular simulations through multi-level parallelism from laptops to supercomputers, *SoftwareX* **2015**, *1*, 19-25.

[9] D. Van Der Spoel, E. Lindahl, B. Hess, G. Groenhof, A. E. Mark, H. J. Berendsen, GROMACS: fast, flexible, and free, *Journal of computational chemistry* **2005**, *26*, 1701-1718.

[10] S. Páll, M. J. Abraham, C. Kutzner, B. Hess, E. Lindahl, in *International conference on exascale applications and software*, Springer, **2014**, pp. 3-27.

[11] S. Pronk, S. Páll, R. Schulz, P. Larsson, P. Bjelkmar, R. Apostolov, M. R. Shirts, J. C. Smith, P. M. Kasson, D. Van Der Spoel, B. Hess, E. Lindahl, GROMACS 4.5: a high-throughput and highly parallel open source molecular simulation toolkit, *Bioinformatics* **2013**, *29*, 845-854.

[12] E. Lindahl, B. Hess, D. Van Der Spoel, GROMACS 3.0: a package for molecular simulation and trajectory analysis, *Molecular modeling annual* **2001**, *7*, 306-317.

[13] H. J. Berendsen, D. van der Spoel, R. van Drunen, GROMACS: A message-passing parallel molecular dynamics implementation, *Computer physics communications* **1995**, *91*, 43-56.

[14] B. Hess, C. Kutzner, D. Van Der Spoel, E. Lindahl, GROMACS 4: algorithms for highly efficient, load-balanced, and scalable molecular simulation, *Journal of chemical theory and computation* **2008**, *4*, 435-447.

[15] R. Pascazio, F. Zaccaria, B. Van Beek, I. Infante, Classical force-field parameters for CsPbBr3 perovskite nanocrystals, *J. Phys. Chem. C* **2022**, *126*, 9898-9908.

[16] van Beek B., Auto-FOX, Published online May **2023**. doi:10.5281/zenodo.7919377.

[17] S. Cosseddu, I. Infante, Force field parametrization of colloidal CdSe nanocrystals using an adaptive rate Monte Carlo optimization algorithm, *Journal of Chemical Theory and Computation* **2017**, *13*, 297-308.

[18] U. Essmann, L. Perera, M. L. Berkowitz, T. Darden, H. Lee, L. G. Pedersen, A smooth particle mesh Ewald method, *The Journal of chemical physics* **1995**, *103*, 8577-8593.

[19] G. Bussi, D. Donadio, M. Parrinello, Canonical sampling through velocity rescaling, *The Journal of chemical physics* **2007**, *126*.

[20] M. Parrinello, A. Rahman, Polymorphic transitions in single crystals: A new molecular dynamics method, *Journal of Applied physics* **1981**, *52*, 7182-7190.
[21] J. D. Yesselman, D. J. Price, J. L. Knight, C. L. Brooks III, MATCH: An atom-typing toolset for molecular mechanics force fields, *Journal of computational chemistry* **2012**, *33*, 189-202.
[22] K. Vanommeslaeghe, E. Hatcher, C. Acharya, S. Kundu, S. Zhong, J. Shim, E. Darian, O. Guvench, P. Lopes, I. Vorobyov, A. D. Mackerell, CHARMM general force field: A force field for drug-like molecules compatible with the CHARMM all-atom additive biological force fields, *Journal of computational chemistry* **2010**, *31*, 671-690.
